# Efficacy of Novel Aminooxyacetic Acid Prodrugs in Colon Cancer Models: Towards Clinical Translation of the Cystathionine β-Synthase Inhibition Concept

**DOI:** 10.3390/biom11081073

**Published:** 2021-07-21

**Authors:** Mark R. Hellmich, Celia Chao, Katalin Módis, Ye Ding, John R. Zatarain, Ketan Thanki, Manjit Maskey, Nadiya Druzhyna, Ashley A. Untereiner, Akbar Ahmad, Yu Xue, Haiying Chen, William K. Russell, Jianmei Wang, Jia Zhou, Csaba Szabo

**Affiliations:** 1 Department of Surgery, University of Texas, Medical Branch, Galveston, TX 77555, USA; cechao@utmb.edu (C.C.); kamodis@utmb.edu (K.M.); jrzatara@utmb.edu (J.R.Z.); kkthanki@gmail.com (K.T.); mamaskey@utmb.edu (M.M.); 2 Department of Anesthesiology, University of Texas, Medical Branch, Galveston, TX 77555, USA; nadruzhy@utmb.edu (N.D.); ashley.untereiner@gmail.com (A.A.U.); akahmad@utmb.edu (A.A.); 3 Department of Pharmacology and Toxicology, University of Texas, Medical Branch, Galveston, TX 77555, USA; dingye515@outlook.com (Y.D.); yuxue1@utmb.edu (Y.X.); haichen@utmb.edu (H.C.); 4 Department of Biochemistry and Molecular Biology, University of Texas, Medical Branch, Galveston, TX 77555, USA; wirussel@utmb.edu; 5 College of Pharmacy, University of North Texas Health Science Center, Fort Worth, TX 76107, USA; jianmei.wang@unthsc.edu; 6 Chair of Pharmacology, Section of Medicine, University of Fribourg, CH-1700 Fribourg, Switzerland

**Keywords:** hydrogen sulfide, anticancer, biomarker, prodrug

## Abstract

Upregulation of hydrogen sulfide (H_2_S) biosynthesis, at least in part related to the upregulation of cystathionine β-synthetase (CBS) in cancer cells, serves as a tumor-promoting factor and has emerged as a possible molecular target for antitumor drug development. To facilitate future clinical translation, we have synthesized a variety of novel CBS-targeting, esterase-cleavable prodrugs based on the structure of the prototypical CBS inhibitor aminooxyacetic acid (AOAA). The pharmacological properties of these compounds were evaluated in cell-free assays with recombinant human CBS protein, the human colon cancer cell line HCT116, and in vivo using various tumor-bearing mice models. The prodrug YD0251 (the isopropyl ester derivative of AOAA) was selected for detailed characterization. YD0251 exhibits improved antiproliferative efficacy in cell culture models when compared to AOAA. It is up to 18 times more potent than AOAA at suppressing HCT116 tumor growth in vivo and is effective when administered to tumor-bearing mice either via subcutaneous injection or oral gavage. Patient-derived xenografts (PDTXs) with higher levels of CBS protein grew significantly larger than tumors with lower levels, and YD0251 treatment inhibited the growth of PDTXs with elevated CBS, whereas it had no significant effect on PDTXs with low CBS protein levels. The toxicity of YD0251 was assessed in mice subjected to subchronic administration of supratherapeutic doses the inhibitor; no significant alteration in circulating markers of organ injury or histopathological alterations were noted, up to 60 mg/kg/day × 5 days. In preparation to a future theranostic concept (to match CBS inhibitor therapy to high-CBS expressors), we identified a potential plasma marker of CBS-expressing tumors. Colon cancer cells produced significant levels of lanthionine, a rare metabolic intermediate of CBS-mediated H_2_S biosynthesis; forced expression of CBS into non-transformed epithelial cells increased lanthionine biogenesis in vitro and in vivo (measured in the urine of tumor-bearing mice). These current results may be useful to facilitate the translation of a CBS inhibition-based antitumor concept into the clinical space.

## 1. Introduction

Twenty-five years ago, Abe and Kimura proposed that the endogenous gaseous mediator hydrogen sulfide (H_2_S) could function as a physiological neuromodulator in the brain [1]. Subsequent studies by them and others have established important physiological roles for H_2_S in a variety of biological processes involving the nervous, cardiovascular, gastrointestinal, endocrine, and immune systems [2,3,4,5,6]. The principal sources of endogenously synthesized H_2_S in mammalian cells are the two transsulfuration pathway enzymes CBS and cystathionine γ-lyase (CSE), and a third enzyme, 3-mercaptopyruvate sulfurtransferase (3-MST) [2,3,4,5,6,7,8].

Given its importance in normal physiology, it is not surprising that dysregulation of H_2_S homeostasis is implicated in the pathophysiology of many diseases, with some illnesses including atherosclerosis, colitis, type 2 diabetes, psoriasis, and hepatocellular carcinoma exhibiting local and/or systemic suppression of physiological levels of H_2_S, while other maladies (e.g., various forms of shock and inflammation, and Down syndrome) are associated with an aberrant upregulation of H_2_S production [8,9,10,11,12,13,14,15].

Cancers of the colon [16], ovaries [17], prostate [18], and breast [19] belong to the group of human diseases associated with a pathological upregulation of CBS and CBS-dependent H_2_S production. In 2013, we first reported the aberrant upregulation of CBS in human colon tumor tissue and colon cancer-derived cell lines and demonstrated that inhibition of CBS expression and/or activity had antitumor effects [16]. We went on to show that CBS expression was increased in human adenomatous polyps and that experimental upregulation of CBS in a premalignant colonic epithelial cell line, NCM356, caused extensive metabolic reprogramming and induction of an invasive tumorigenic phenotype, supporting the conclusion that enhanced CBS activity not only contributes to the progression and the spread of established cancer, but also promotes colon carcinogenesis [20]. 

More recently, we have demonstrated that resistance to the chemotherapeutic agent 5-fluorouracil was associated with increased CBS protein levels and that inhibition of CBS activity restored cancer cell chemosensitivity [21]. Given this growing body of experimental and clinical evidence for the role of H_2_S as a pro-cancer bioenergetic, proliferative, and survival factor [16,17,18,19,20,21,22,23,24,25,26], we have pursued the development of novel inhibitors of cancer cell CBS activity as potential future anti-cancer therapies.

To date, we have conducted medium-throughput screening of various clinically approved and pharmacologically well-characterized small molecules and identified several compounds that may be potentially suitable for repurposing as CBS inhibitors [27]. We have also synthesized and tested derivatives of the prototypical CBS inhibitor aminooxyacetate (AOAA) in different cellular and animal models of colon cancer [16,21,28]. The studies presented herein represent a continuation of these efforts, with a focus towards the potential future translation of CBS-inhibitors for the treatment of colorectal cancers.

## 2. Materials and Methods

### 2.1. Materials

All chemicals were obtained from Sigma-Aldrich, St. Louis, MO, USA, unless otherwise stated. For the synthesis of the various AOAA prodrugs, all commercially available starting materials and solvents were reagent grade and used without further purification. Reactions were performed under a nitrogen atmosphere in dry glassware with magnetic stirring. Preparative column chromatography was performed using silica gel 60, particle size 0.063–0.200 mm (70–230 mesh, flash). Analytical TLC was carried out on silica gel 60 F254 plates (Merck, Darmstadt, Germany). Nuclear magnetic resonance (NMR) spectra, recorded on a Bruker 300 (^1^H, 300 MHz; ^13^C, 75 MHz) spectrometer (Billerica, MA, USA), were used to confirm the identity of the synthesized compounds. ^1^H and ^13^C NMR spectra (see supplementary) were recorded with tetramethylsilane (TMS) as an internal reference. Chemical shifts downfield from TMS were expressed in ppm, and J values were given in Hz. High-resolution mass spectra (HRMS) were obtained using a Thermo Fisher LTQ Orbitrap Elite mass spectrometer. Parameters included the following: nano electrospray ionization (ESI) voltage of 1.8 kV, capillary temperature of 275 °C, and resolution of 60,000; ionization was achieved by positive mode. NMR spectra of the synthesized compounds are shown in the Supporting Information section.

**Figure 1:**
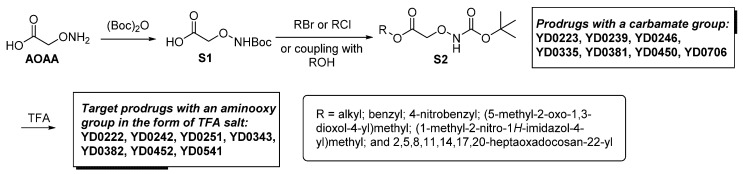
Synthetic routes applied to synthesize novel AOAA derivative prodrugs, with structures shown in Table 1.

One of the design goals for the new AOAA-based prodrugs was to improve lipophilicity (i.e., increase ClogP values) relative to AOAA to enhance cellular uptake according to Lipinski’s “Rule of Five”. Key synthetic methods used to generate the representative prodrugs are shown in Figure 1. The amino group of AOAA was protected with Boc to give the key intermediate S1. The substitution reaction between the acid of S1 and various brominated/chlorinated reagents generated different S2 esters, including the prodrugs with a carbamate group. 

Finally, the prodrugs with an aminooxy group in the form of trifluoroacetic acid (TFA) salt were obtained by Boc-deprotection with TFA in CH_2_Cl_2_ (*v*/*v* 1:4). 

**Figure 2:**
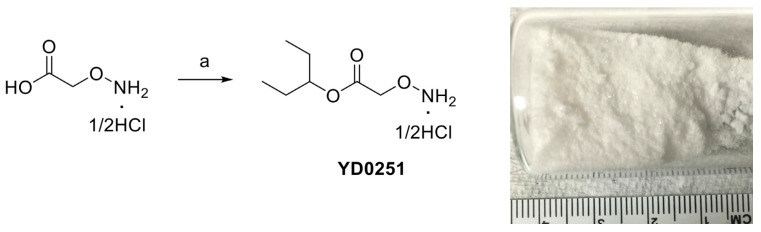
Streamlined synthesis of YD0251 suitable for scale-up at the gram to kilogram level. Reagents and conditions: (a) 3-pentanol, SOCl_2_, rt, 2 h, 100 °C, 16 h.

Since much of the follow-up efficacy and safety work focused on YD0251 (the isopentyl ester of AOAA), a second, more facile chemical synthetic route was developed suitable for scale-up from gram to kilogram quantities of prodrug (Figure 2, left panel). In this method, SOCl_2_ (2.18 g, 18.30 mmol) was added to a mixture of carboxymethoxylamine hemihydrochloride (1.0 g, 9.15 mmol) in 3-pentanol (5 mL) at 0 °C. The resulting mixture was stirred, first at room temperature for 2 h and then for an additional 16 h at 100 °C (the reaction progress was monitored by NMR). The solvent was removed under reduced pressure to achieve the desired product YD0251 as a white solid (1.41 g, 86%), which was recrystallized from 3-pentanol (5 mL) (Figure 2, right panel), washed with 10% EtOAc/hexane solution (25 mL), and dried under vacuum. TLC: Rf = 0.4~0.5 (20% EtOAc/hexanes). ^1^H-NMR (300 MHz, D_2_O): δ 4.80 (m, 1H), 4.66 (s, 2H), 1.54 (m, 4H), 0.77 (t, *J* = 7.5 Hz, 6H). 13C-NMR (75 MHz, D_2_O): δ 169.6, 80.79, 70.58, 25.67, 25.67, 8.73, 8.73. HRMS Calcd for C_7_H_16_NO_3_: [M + H]^+^ 162.1130; found 162.1124.

The structures of the successfully synthesized prodrug test compounds (together with the reference compound AOAA) are shown in Table 1. YD0382 and YD0452 were designed as potentially hypoxia-activatable prodrugs based on their particular ester structure.

### 2.2. Measurement of the Catalytic Activity of Recombinant Human CBS

Full-length recombinant human CBS was produced as described [28]. H_2_S production by CBS was measured using the 7-azido-4-methylcoumarin (AzMC) method [28] in a 96-well black plate format. The total assay volume was 200 μL per well and consisted of 170 μL of reaction solution containing final concentrations of 200 mM Tris HCl (pH 8.0), 5 μM pyridoxal 5′-phosphate (PLP), 10 mM glutathione, and 0.5 mg/mL BSA; 10 µL test compound; 10 µL CBS (5 μg); and 10 μL of a mixture of AzMc (10 μM) and the CBS substrates L-cysteine and homocysteine (each at 2.5 mM final concentration). The reaction mixture was incubated at 37 °C for 1 h, and AzMc fluorescence read at 450 nm (λex = 365 nm). Percent inhibition of CBS activity of each test compound (at 100 µM) is shown in Table 1 as the mean value of *n* = 3 determinations.

### 2.3. Effect of Test Compounds on Cell Proliferation and Viability 

The human colorectal carcinoma cell line, HCT116, was cultured in McCoy’s 5A medium supplemented with 10% FBS, 100 IU/mL penicillin, and 100 mg/mL streptomycin as described [16]. Cells were grown in a 37 °C, 5% CO_2_ atmosphere. For assessment of cell proliferation, the xCELLigence Real-time Cell Analyzer (Agilent Technologies, Santa Clara, CA, USA) was used, as described [16]. Briefly, HCT116 cells were cultured until approximately 70% confluence. Cells were detached by Trypsin–EDTA and re-suspended in fresh culture media at a concentration of 60,000 cells/mL. Then, 100 µL of cell suspension was added to each well (6000 cells/well) of an E-plate after recording the background with 100 µL of cell culture media per well. The E-plates are specially designed 96-well microtiter plates containing interdigitated microelectrodes to non-invasively monitor the cell proliferation by measuring the relative change in the electrical impedance of the cell monolayer, a unitless parameter named the “cell index”. Once plated, the cells were treated either with vehicle (i.e., test compound diluent) or different concentrations of the test compounds (10–1000 µM). The change in electrical impedance (i.e., proliferation rate) was monitored for 48 h. The percent inhibitions of vehicle-treated cell cultures for each concentration of the test compound (at 10, 30, 100, and 300 µM) at 48 h are shown in Table 1 as the mean values of *n* = 3 determinations to compare the effects of YD0251 and 5-fluorouracil (5-FU) on the viability of control and multidrug-resistant HCT116 cells, generated and characterized as previously described [21]. Cells were incubated either with various concentrations of YD0251 or 5-FU for 24 h, and mitochondrial-dependent conversion of MTT to formazan was measured as described [16,21].

### 2.4. Ex Vivo Assessment of Tumor and Liver CBS Activity Following In Vivo Treatment with Test Compounds

The animal studies conducted in the current paper were approved by UTMB’s IACUC and were conducted using protocols 890102I and 8901012J (In vivo studies in GI cancer growth, approved 1 January 2016 and valid until 31 December 2021). 

Nu/nu Balb/C mice (4–6 per test compound) were implanted with HCT116 cells subcutaneously as described [16]. When tumor size reached ~150 mm^3^, animals were treated either with vehicle or a test compound (2.5 mg/kg) via s.q. injection every 6 h for 24 h (total dose 10 mg/kg). Following treatment, tumor and liver tissues were harvested and protein homogenates prepared for ex vivo CBS activity measurements using the AzMC-based H_2_S production assay as described [28]. Inhibition of CBS activity was calculated as follows: ((vehicle-treated CBS activity—AOAA or prodrug-treated CBS activity)/vehicle-treated CBS activity) × 100. Results are shown in Table 1. Each value is the mean of *n* = 3 determinations.

### 2.5. Assessment of the Efficacy of YD0251 and AOAA in Mice Bearing HCT116 Subcutaneous Xenografts

Tumors were initiated by injecting Nu/nu Balb/C mice with HCT116 cells (1 × 10^6^ cells/injection) subcutaneously in the right or left rear flank. Then, 7–10 days after tumor initiation, animals were treated either with vehicle or test compounds via s.q. injection or oral gavage at different doses once a day for 20 days. Tumor size (volume) was measured transcutaneously with a caliper every 2–3 days as described [16]. Results are presented as the mean ± SEM values of *n* = 7–9 determinations.

### 2.6. Assessment of the Efficacy of YD0251 in Mice Bearing Subcutaneous Patient-Derived Tumor Xenografts (PDTXs)

Freshly resected colorectal cancer tissue was collected under an IRB-approved protocol as part of a discarded tissue biobanking protocol. At the time of tissue collection, the establishment of this protocol did not require specific patient informed consent. Cancer tissues were dissected by a surgical pathologist and immediately placed into a sterile tube containing cold DMEM supplemented with 200 U/mL penicillin, 200 μg/mL streptomycin, 0.25 μg/mL amphotericin B, and 50 μg/mL gentamicin (Invitrogen; Carlsbad, CA, USA). After several washing steps in this medium, a small piece was minced into ~2–3 mm^3^ pieces under sterile conditions for xenografting into the flanks of 2 or 3 Nu/nu Balb/C mice. This initial engraftment was designated “passage zero” (P0). The studies described here used PDTXs at passages 3 to 5 (i.e., P3 to P5). 

To test the effects of the prodrugs on PDTX growth in vivo, once the tumor measured ~100 mm along 2 axes, animals were treated daily for up to 3 weeks either with vehicle or test compounds at a dose of 6 mg/kg (100 μL, subcutaneous injection). Tumor growth was monitored by transcutaneous measurements with a caliper every 2–3 days as described [16]. PDTXs from 6 different patients were used. The level of CBS protein expression in each PDTX was determined by Western blotting as described [16].

### 2.7. Assessment of the Safety and Tolerability of YD0251 in Mice

Balb/C mice were subjected to intraperitoneal injections of YD0251 (total daily doses: 2 mg/kg/day, 6 mg/kg/day, 20 mg/kg/day, and 60 mg/kg/day) for 7 consecutive days. On the morning of the eighth day, the experiment was terminated, blood was collected and analyzed using the Vetscan VS2 Chemistry Analyzer (Abaxis/Zoetis, Parsippany, NJ, USA), and histopathological assessment of the liver and kidney was conducted in a blinded fashion as described [28]. The data on plasma markers are presented as mean ± SEM of *n* = 5 mice per group; histological images are presented as representative examples.

### 2.8. Sample Preparation and Detection of Transsulfuration Pathway Metabolites by Mass Spectrometry

Sample preparation: 20 μL of working internal standard solution was added to 150 μL of each urine sample or cell extract. An additional 90 μL of cold acetonitrile with 0.1% FA was added, and the resulting solution vortexed for 5 min. After vortexing, tubes were centrifuged for 5 min at 13,000 rpm, and 200 μL of each supernatant was transferred to an HPLC glass vial with a glass insert for analysis. Liquid chromatography–mass spectrometry (LCMS) analysis was performed on a Sciex 6500 QTRAP (Framingham, MA, USA) coupled with an Agilent 1260 ultra-high pressure liquid chromatography (UHPLC) system. Separation was accomplished by using a Waters Atlantis Hillic Silica column (150 mm × 2.1 mm, 5 μM). The pump flow rate was set at 0.5 mL/min with the gradient: 0–1.5 min, 95% B; 3–10.5 min, 80% B; 11–13 min, 60% B; 13.1–20 min, 95% B. Mobile phase A composition consisted of 1000 mL water + 10 mL of 1 M ammonium formate + 2 mL formic acid. Mobile phase B consisted of 950 mL acetonitrile + 40 mL water + 10 mL of 1 M ammonium formate + 2 mL formic acid. 

The transsulfuration pathway metabolites were separated by UHPLC and monitored using scheduled multiple reaction monitoring (MRM) mode [29]. Heavy stable isotope-labeled internal standards for cystathionine, serine, and homocysteine were used for internal calibration.

### 2.9. Statistics

All the results were expressed as mean ± standard error (SEM) of at least 3 independent experiments. Differences among means were considered significant if *p* ≤ 0.05. Normal distribution was determined using either the Shapiro–Wilk test or D’Agostino and Pearson test. To analyze normally distributed data, we used either an unpaired t-test (two-tailed), one-way ANOVA, or 2-way ANOVA, with Tukey’s multiple comparison post-hoc tests. For analyses of non-parametric data, we used the Mann–Whitney test (two-tailed). Statistical calculations were performed using GraphPad Prism 9 (GraphPad Software Inc., San Diego, CA, USA).

## 3. Results and Discussion

### 3.1. AOAA-Based Prodrugs Are Weak CBS Inhibitors in the Absence of Bioactivation

We initially evaluated the relative potency of each AOAA-based prodrug against the reference compound (i.e., authentic AOAA) in a cell-free assay using purified recombinant human CBS enzyme [27]. H_2_S production was measured by the AzMC method, with the results summarized in Table 1. We expected that, unlike AOAA, the prodrug structures would be too large to tightly interact with the PLP binding pocket of CBS without esterase-mediated bioactivation. Consequently, their inhibitory potency would be weaker than AOAA. Indeed, simply replacing the hydroxy group of AOAA with a methyl ester (YD0171) reduced the percent inhibition of recombinant CBS-mediated H_2_S production from ~92% for the reference compound to only ~42% for the prodrug (Table 1). 

In all, 10 of the 15 AOAA-based prodrugs exhibited less than 10% inhibition of recombinant CBS activity when used at a concentration of 100 μM (Table 1), demonstrating that in the absence of enzymatic cleavage, the prodrug structures are generally poor CBS inhibitors.

### 3.2. Bioactivation Enhances the Inhibitory Activity of the AOAA-Based Prodrugs YD0171 and YD0251

To assess the role of bioactivation in prodrug-mediated CBS inhibition, we incubated AOAA, YD0171, or YD0251 (final concentrations, 30 μM) in mouse plasma or a cytosolic extract from HCT116 cancer cells for up to 2 h at 37 °C. We selected these biofluids because it is well known that rodent plasma contains relatively high levels of carboxylesterase activity when compared to other species [30,31,32], and therefore it would be a good positive control. In addition, the cancer cell extract is relevant to our overall goal of developing antitumor therapeutics. 

**Figure 3:**
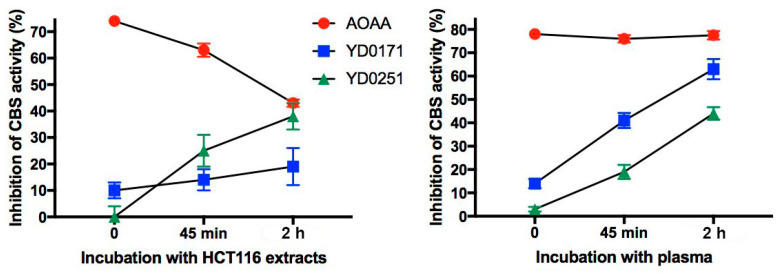
Comparison of the CBS inhibitory activity of AOAA, YD0251, or YD0171 before and after incubation in cytosolic extracts of HCT116 cells (left panel) or mouse plasma (right panel). Each compound was added to the biofluid to give a final concentration of 30 µM. At time 0 min, 45 min, or 2 h, the recombinant CBS was added and CBS-mediated H_2_S production was measured using AzMC. Inhibition of CBS activity was calculated as follows: ((vehicle-treated CBS activity—AOAA or prodrug-treated CBS activity)/vehicle-treated CBS activity) × 100. Data points are mean values ± SEM from 3 independent experiments.

At the start of the incubation period (i.e., time = 0) and at 45 min and 2 h (120 min), the recombinant CBS protein was added to the biofluid/AOAA or prodrug mixtures, and H_2_S production measured using AzMC as described above. Vehicle-treated control plasma and cytosolic extracts “spiked” with recombinant CBS at each time point were used to determine maximum CBS activity and to calculate the percent inhibition of CBS activity by each test compound. At the initial time point (t = 0), the HCT116 cytosolic extract and mouse plasma samples containing AOAA exhibited between 70% and 80% inhibition of vehicle-treated control CBS activity, respectively (Figure 3). The inhibitory efficacy of AOAA incubated in the cytosolic extract declined over time, presumably due to either metabolic inactivation or perhaps binding to non-CBS proteins, whereas its inhibitory potency remained stable in plasma (Figure 3). 

The high level of CBS inhibitory activity at time zero was expected for AOAA given the fact that it does not require esterase-mediated bioactivation. In contrast, the cytosolic extracts and plasma samples containing the prodrugs exhibited little to no CBS inhibitory activity at time zero; <15% for YD0171 and ~0% for YD0215, but showed gradually increasing inhibition at 45 min and 2 h, consistent with esterase-mediated bioactivation (Figure 3). As expected, both compounds were activated in mouse plasma, with YD0171 inhibiting 60% of control CBS activity by 2 h and YD0251 inhibiting ~40% (Figure 3). However, in the cancer cell cytosolic extracts, the inhibitor efficacy of YD0251 was almost two times greater than that of YD0171 at the final timepoint (Figure 3), suggesting that YD0251 may be preferable to YD0171 for targeting tumor CSB activity. 

### 3.3. AOAA Prodrugs Show Enhanced Antiproliferative Efficacy Compared to AOAA In Vitro

HCT116 cells were treated over a 48-h time course either with vehicle (100% growth control) or different concentrations of AOAA or an AOAA-based prodrug (i.e., 10, 30, 100, 300, and 1000 µM). In designing the different prodrug structures, we reasoned that increasing the lipophilicity (i.e., ClogP value) of AOAA with the addition of ester- and/or amide-linked functional groups (i.e., R-groups) would enhance the cellular uptake of the novel structures and thus increase their relative inhibitor potencies when compared to the unmodified reference compound. For example, two prodrugs with amide-linked carbamate groups (YD0381 and YD0239) were found to inhibit HCT116 cell proliferation by ~3% and 8%, respectively, at a concentration of 30 μM, and by ~41% at 300 μM (Table 1). By comparison, the reference compound, AOAA, had no effect on cell replication at the lower concentrations and only inhibited proliferation by ~36% at 300 μM (Table 1). The free aminooxy forms of YD0381 (i.e., YD0382) and YD0239 (i.e., YD0242) were also not effective inhibitors at low concentrations, but induced more inhibition than their carbamate forms at 300 μM, inhibiting ~50% and ~65% of vehicle-treated cell proliferation, respectively (Table 1). This observation is not surprising given that, similarly to AOAA, the free aminooxy groups of these compounds are protonated (NH_3_^+^) at physiological pH, which, relative to their carbamate forms, reduces their lipophilicity and likely slows cellular uptake. However, once inside the cell, YD0382 and YD0242 only require enzyme-mediated cleavage of one ester-linked functional group to generate AOAA, which may account for their increased inhibitory activity at the highest concentration.

**Figure 4:**
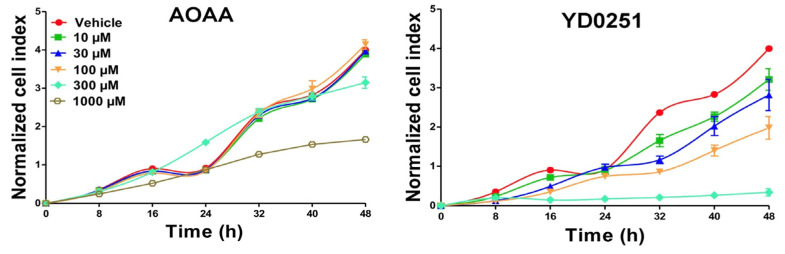
Effects of different concentrations of AOAA (left panel) and YD0251 (right panel) on HCT116 cell proliferation In Vitro. HCT116 cells were treated either with vehicle (i.e., test compound diluent) or different concentrations of the test compounds (10–1000 µM), and proliferation was monitored for 48 h. Proliferation rates were monitored using the xCELLigence Real-time Cell Analyzer as described [17]. Data are shown as mean of *n* = 6 technical replicates at each time point.

Of all the novel compounds analyzed in the HCT116 cell proliferation assay, YD0171 and YD0251 showed the greatest potency, inhibiting cell growth at a concentration as low as 10 μM (Table 1). As shown in Figure 4, YD0251 inhibited HCT116 cell proliferation at all concentrations tested by 32 h, with greater than 50% inhibition of vehicle control proliferation observed at 100 μM. At a concentration of 300 μM, YD0251 exerted a cytostatic effect on cell proliferation across the 48-h time course (Figure 4). By comparison, it took a 10-fold higher concentration of AOAA (i.e., 1000 μM) to achieve ~50% inhibition of HCT116 cell proliferation at 32 h (Figure 4).

Thus, these data indicate that YD0251 is more potent than AOAA in inhibiting CBS in cell-based systems (although it is less potent than AOAA in the isolated CBS enzyme). These properties of YD0251 are similar to YD0171 [28], an earlier-generation CBS inhibitor prodrug, previously synthesized and characterized by us in various colon cancer cell lines in vitro and colon cancer models in vivo. The data presented above are consistent with the hypothesis that YD0251 is converted to AOAA by intra- and extracellular esterases, and ultimately, it is AOAA that is responsible for CBS inhibition. While the prodrug strategy is useful to improve cell uptake and thus cell-based potency, the selectivity of YD0251 to CBS is identical to the selectivity of AOAA, i.e., not particularly selective. Indeed, AOAA is known to also inhibit CSE (another H_2_S-producing enzyme), as well as a variety of PLP-dependent enzymes that are unrelated to H_2_S biosynthesis (e.g., GABA-transferase and various transaminases) [26]. These additional actions, on a variety of additional enzymes, may in fact contribute to the cellular or in vivo effects of YD0251.

### 3.4. Ex Vivo Assessment of Tumor and Liver CBS Activity after In Vivo Treatment with AOAA Prodrugs

In a separate subset of in vivo experiments, tumor xenografts were initiated by injecting 1 × 10^6^ HCT116 cells subcutaneously in the right or left flank of Nu/nu Balb/C mice. When the tumor reached a size of ~150 mm^3^, the animals were acutely treated either with vehicle or the different test compounds (i.e., prodrugs) at a dose of 2 mg/kg, injected subcutaneously every 6 h for 24 h (total dose; 10 mg/kg). In total, 4–6 mice were used to evaluate each test compound. At the end of the in vivo treatment period, tumor and liver tissue homogenates were prepared for ex vivo CBS activity assays using the AzMC method. The data, expressed as percent inhibition of vehicle-treated control CBS activity, are shown in the last two columns of Table 1.

Treating mice with a total of 10 mg/kg of AOAA over 24 h had no effect on the tumor CBS activity ex vivo (Table 1). In other words, the amount of CBS activity measured ex vivo in tumor homogenates from AOAA-treated mice was equivalent to the activity measured in the tumor homogenates from vehicle-treated mice, suggesting that the HCT116 tumor tissue took up and/or retained relatively little AOAA during the acute in vivo treatment period. By comparison, the liver homogenates from AOAA-treated mice exhibited ~36% less CBS activity than the livers from vehicle-treated mice. YD0242, YD0541, and YD0706 behaved similarly to AOAA in that they inhibited vehicle-treated liver CBS activity, but did not inhibit tumor CBS activity, as measured by tissue homogenate H_2_S production (Table 1). Three test compounds (YD0343, YD0382, and YD0452) had no effects on either tumor or liver ex vivo H_2_S production; several other test compounds (YD0246, YD0251, and YD0335), inhibited both tumor and liver CBS activity. On average, the inhibition of liver CBS by these compounds was ~15% greater than that measured in the corresponding tumor tissue homogenates. Greater inhibition of liver CBS than tumor activity was observed with 9 of the 15 test compounds (Table 1), suggesting that the liver cells are more efficient than cancer cells at taking up and/or bioactivating most of the prodrug structures. Only two test compounds (YD0171 and YD0239) were more effective at inhibiting tumor CBS activity than liver using the acute dosing regimen. Tumor homogenates from YD0171-treated mice exhibited ~68% less CBS activity than tumor homogenates from vehicle-treated control animals, whereas liver homogenates from the same animals showed only an ~15% reduction of control liver CBS activity (Table 1). Similarly, YD0239 reduced tumor ex vivo CBS activity by ~63% relative to vehicle-treated control tumors, while exerting no inhibitory activity on liver CBS.

### 3.5. YD0251 Exhibits Improved Antitumor Potency In Vivo When Compared to AOAA 

**Figure 5:**
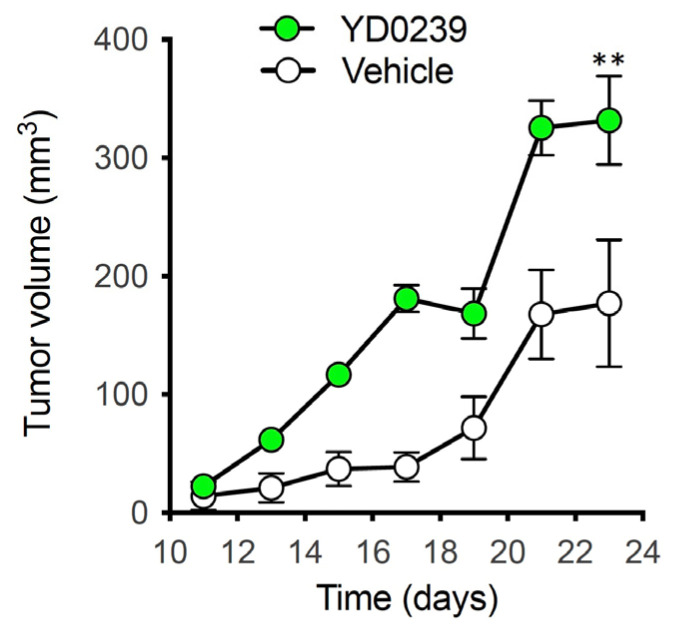
Effects of YD0239 on HCT116 tumor xenograft growth In Vivo. HCT116 tumor-bearing Nu/nu Balb/C mice were treated with either vehicle or YD0239 (6 mg/kg/daily) via s.q. injection once a day for 24 days. Tumor size (volume) was measured transcutaneously with a caliper every 2–3 days. (*n* = 6 mice/group, ** *p* < 0.01 showing significant enhancement of tumor size).

To assess the antitumor efficacy of selected AOAA-based prodrugs with long-term dosing (i.e., 14–28 days), we initially focused on YD0251 and YD0239 because these compounds were significantly more potent than AOAA in inhibiting HCT116 cell proliferation in vitro (Table 1). However, unexpectedly, a preliminary in vivo evaluation of YD0239 showed a significant (*p* < 0.005) increase in the size of HCT116 tumor xenograft growth in mice treated for 24 days (6 mg/kg/daily), when compared to vehicle-treated animals (Figure 5). The mechanism behind this enhancement is unclear but may be due to a hitherto unknown secondary enzymatic target of this particular molecule.

**Figure 6:**
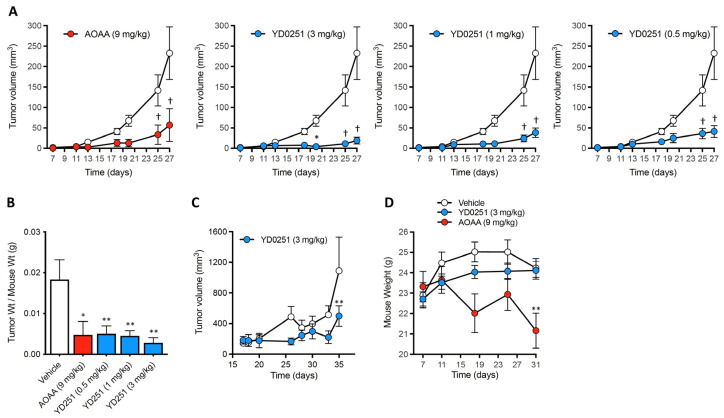
YD0251 is more potent than AOAA at inhibiting HCT116 tumor xenograft growth In Vivo. The compounds were administered daily at the indicated doses by subcutaneous injection (**A**,**B**) and oral gavage (**C**). Data are expressed as ratio of tumor weight to mouse body weight at harvest following 21 days of treatment. YD0251 did not affect the weight of the animals over the course of the experiments, while AOAA induced a significant weight loss by Day 31 (**D**). (*n* = 6 mice/group, * *p* < 0.05, † *p* < 0.05, ** *p* < 0.01).

In the remainder of our studies, we focused on the further characterization of the antitumor properties of YD0251. Our results demonstrated that YD0251 is 3 to 18 times more potent than AOAA in vivo. Treating HCT116 tumor-bearing mice via subcutaneous injection with either a daily dose of 0.5, 1, or 3 mg/kg YD0251 markedly inhibited the rate of xenograft growth (Figure 6A). Importantly, the lower doses of YD0251 were as efficacious as treating with 9 mg/kg of AOAA (Figure 6A). Furthermore, YD0251 at doses of 1 and 3 mg/kg effectively inhibited in vivo tumor growth both in the subcutaneous injection and the oral gavage therapeutic regimens (Figure 6A,B).

### 3.6. YD0251 Inhibits Patient-Derived Tumor Xenograft Growth 

Next, we tested the growth inhibitory efficacy of YD0251 using patient-derived tumor xenografts (PDTXs). Low-passage PDTXs have emerged as an important preclinical model for oncological drug discovery because they maintain the histological features, gene expression profile, and mutation status of the patient’s original tumor tissue [33]. 

**Figure 7:**
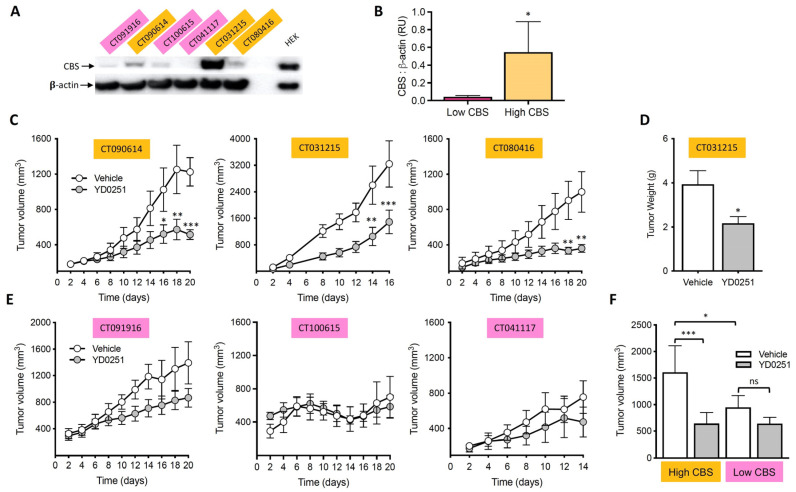
In Vivo efficacy of YD0251 on the growth of PDTX in nude mice. (**A**) Example Western blot showing varying levels of CBS protein in PDTXs from 6 different patients. (**B**) Comparison of the relative levels of CBS protein expression in the various tumors. (**C**–**E**) The effect of YD0251 (subcutaneous dosing, 6 mg/kg/day) is shown in nude mice bearing human patient-derived xenografts exhibiting high (**C**,**D**) or low (**E**) CBS expression. Note that YD0251 was efficacious in 6/8 patients; it was efficacious in the PDTX’s of all high CBS expressors and in 50% of PDTX’s of low CBS expressors (*n* = mice/group, * *p* < 0.05, ** *p* < 0.01, *** *p* < 0.001, ns: not significant). It was found that 1 PDTX with high CBS levels and 2 PDTXs with low levels of CBS possessed activating mutations of the KRAS gene. The KRAS status of the tumors did not correlate with the growth inhibitory efficacy of YD0251. Overall, high CBS-expressing PDTXs grew faster and the effect of YD0251 was more pronounced in them than in low CBS-expressing PDTXs (**F**).

Colorectal tumors resected from six different patients were used to develop the PDTXs for these studies. Based on their levels of CBS, the six PDTXs were divided into two groups of three (Figure 7A,B). Moreover, the relative growth-inhibitory effect of YD0251 was significantly more pronounced in the high CBS-expressor cohort than in the low CBS-expressor cohort (Figure 7F). 

### 3.7. YD0251 Exerts Antiproliferative Effects in Multidrug-Resistant Colon Cancer Cells

**Figure 8:**
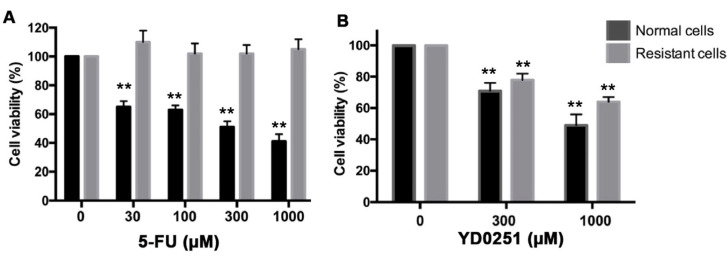
The inhibitory effect of YD0251 on cell viability is largely maintained in multidrug-resistant HCT116 cells. HCT116 cells were maintained in a 5-FU-containing medium for 2 months. Cell assumed a drug-resistant phenotype and lost their responsiveness to 5-FU (**A**), as assessed by the MTT assay 24 h after incubation with various concentrations of YD0251. (**B**) YD0251 treatment resulted in a concentration-dependent reduction in cell viability (*n* = 5, ** *p* < 0.01).

The efficacy of many anticancer drugs diminishes when cancer cells assume a multi-drug resistant phenotype. We have recently established a 5-FU-resistant HCT116 human colon cancer cell line by serial passage in the presence of increasing 5-FU concentrations [21]. These cells also exhibit a partial resistance to an unrelated chemotherapeutic agent, oxaliplatin. The multidrug-resistant cells exhibit a significant increase in the expression of the drug-metabolizing cytochrome P450 enzymes CYP1A2 and CYP2A6 and they also upregulate CBS (as well as another major H_2_S-generating enzyme, 3-MST) [21]. The 5-FU-resistant cells exhibited decreased sensitivity to AOAA but remained sensitive to the antiproliferative effect of benserazide (a recently identified, potentially repurposable CBS inhibitor) [21]. Herein we compared the effects of YD0251 and 5-FU treatments on the viability of multidrug-resistant and parental (normal) HCT116 cell lines. As shown in Figure 8A, 5-FU caused a concentration-dependent decrease in the percentage of viable parental HCT116 but had no effect on the viability of resistant cells. In contrast, YD0251 reduced the viability of both resistant and parental cells (Figure 8B). 

### 3.8. YD0251 at Doses at Which It Exerts Anticancer Effects Does Not Induce Significant Organ Injury

**Figure 9:**
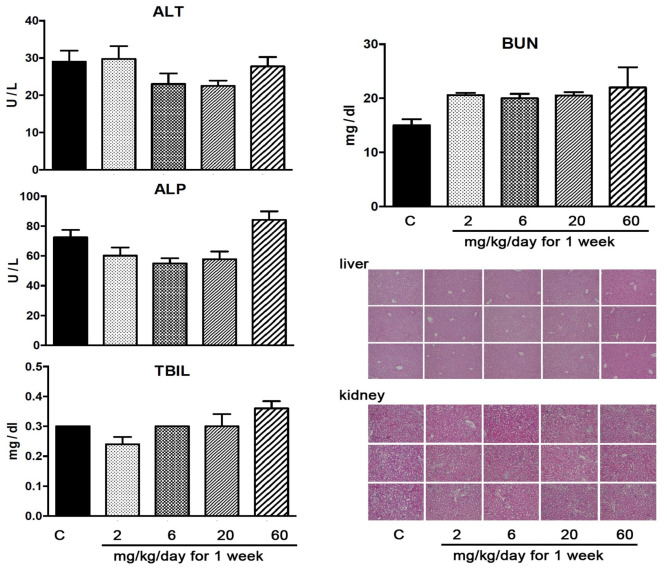
Effect of YD0251 treatment on plasma markers of organ damage and tissue histology. Mice were subjected to intraperitoneal injection of YD0251 in 2 divided doses (total daily doses: 2 mg/kg/day, 6 mg/kg/day, 20 mg/kg/day, and 60 mg/kg/day) for 7 consequent days (1 week). On the morning of the eighth day, the experiment was terminated, and blood was collected and analyzed by the Vetscan system (ALT: alanine aminotransferase; ALP: alkaline phosphatase; TBIL: total bilirubin; BUN: blood urea nitrogen). C: vehicle control group. The mean ± SEM of *n* = 5 mice per group is shown. In addition, there were no detectable histological alterations in any of the groups (representative H&E pictures are shown).

To begin evaluating the potential in vivo toxicity of YD0251, we administered increasing doses to mice via intraperitoneal injection and measured plasma markers of organ damage as well as liver and kidney histology. As shown in Figure 9, no significant changes were noted in either plasma marker or tissue histology in animals receiving up to 60 mg/kg/day of YD0215 for 7 days.

### 3.9. Identification of Lanthionine as a Potential Urinary Biomarker of High CBS-Expressor Tumors

Finally, we tested the theranostic concept that lanthionine [16] might serve as a biomarker for tumors with high levels of CBS expression. Lanthionine is a natural nonproteogenic amino acid, an analogue of cysteine, consisting of two alanine residues crosslinked on their β-carbon atoms by a thioether linkage. The condensation of two molecules of cysteine (β-replacement reaction) produces H_2_S, as well as lanthionine as a side product. Additionally, the other transsulfuration enzyme (CSE) does not contribute to lanthionine production and there are currently no known enzymes that produce lanthionine (other than CBS) [34,35].

**Figure 10:**
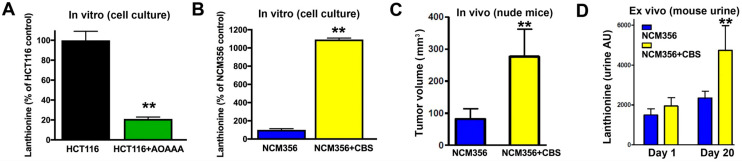
Lanthionine is a potential biomarker of CBS-expressing colon cancer cells. (**A**) Inhibition of CBS suppresses lanthionine production in HCT116 cells. (**B**) Forced overexpression of CBS increases lanthionine production in NCM356 cells. (**C**) Effect of CBS overexpression on NCM356 tumor volume 20 days after implantation. CBS-overexpressing NCM356 cells grow larger tumors than parental cells in nude mice. (**D**) Comparison of urine lanthionine levels NCM356 tumor-bearing mice 1 day and 20 days after tumor cell implantation (*n* = 5, ** *p* < 0.01).

Lanthionine was readily detectable by mass spectrometry in the culture supernatants from HCT116 cells, and inhibition of CBS activity with AOAA reduced lanthionine levels by ~80% (Figure 10A). Furthermore, forced expression of recombinant CBS in NCM356 cells increased lanthionine production over 12-fold and enhanced their tumorigenicity in vivo (Figure 10B,C, respectively). Importantly, we detected significant higher level of lanthionine in the urine of mice bearing the CBS-overexpressing NCM356 tumor, as compared to mice bearing the control NCM356 tumor (Figure 10D). These data provide a rationale to explore the use of plasma or urinary lanthionine as a marker of high CBS-expressor tumors in humans with the goal of identifying patients that could potentially benefit from CBS inhibitory therapy.

## 4. Conclusions

**Figure 11:**
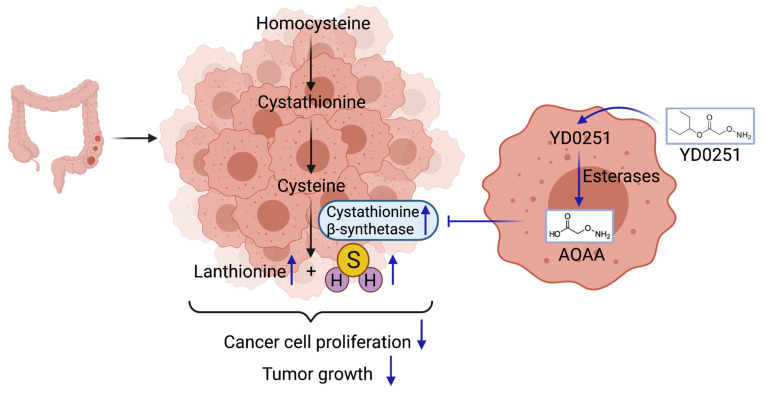
Mechanism of YD0251’s action in colon cancer cells. YD0251, due to its high lipophilicity, readily enters cancer cells. After conversion by esterases, AOAA is released, which in turn inhibits intratumor CBS activity, decreases tumoral H_2_S levels, and exerts antitumor actions in vitro and in vivo. These effects are most pronounced in tumors with higher CBS expression.

In conclusion, the current study identified and characterized the isopropyl ester derivative of AOAA, YD0251, as a novel CBS inhibitor molecule (AOAA prodrug) with significant antiproliferative efficacy in cell culture models of colon cancer (including efficacy in multi-drug resistant variants of human colon cancer cell lines), and with significant antitumor effects in tumor-bearing mice models (Figure 11). 

The current report also shows the diversity in human colon cancer tissues in terms of degree of CBS expression; colon cancers which exhibit high CBS expression proliferate faster when placed in PDTX models than the low CBS-expressor PDTX counterparts, and respond better to the CBS inhibitor’s antiproliferative effect. YD0251 was effective when administered both by subcutaneous injection and oral administration, predicting the oral bioavailability of the molecule. At the doses of YD0251 which were effective in the tumor-bearing mice, as well as at several fold higher doses, the molecule was well tolerated, as evidenced by the fact that repeat administration of relatively high doses did not produce signs of organ injury.

Finally, with an eye towards future theranostic applications (i.e., matching CBS inhibitor therapy to patients with high CBS-expressing tumors), the current study identifies lanthionine (a metabolic intermediate of CBS-mediated H_2_S biosynthesis) as a potential biochemical marker of CBS upregulation. In summary, the current findings provide additional proof-of-concept that CBS is a significant antitumor target in colon cancer and support continued efforts to investigate and eventually clinically develop novel antitumor therapeutics based on CBS inhibition; perhaps in conjunction with the validation of a suitable biomarker (e.g., lanthionine) to identify a high CBS-expressor (and likely a best-responder) colon cancer target population.

## Figures and Tables

**Table 1:** Comparison of the relative inhibitor effects of the different AOAA-based prodrugs to the reference compound, AOAA.

Compound	Category	Structure	CBS Enzyme (% Inhibition at 100 µM)	HCT116 Proliferation (% Inhibition at 10, 30, 100, 300 µM for 48 h)	Tumor Homogen. CBS Activity (% Inhibition)	Liver Homogen. CBS Activity (% Inhibition)
**AOAA**	Reference	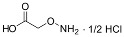	92%	0, 0, 0, 36%	0%	36%
**YD0171**	Prodrug	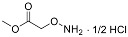	44%	4, 12, 34, 55%	68%	15%
**YD0222**	Prodrug	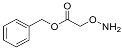	17%	0, 0, 0, 30%	68%	67%
**YD0223**	Prodrug	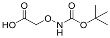	4%	0, 0, 0, 15%	50%	62%
**YD0239**	Prodrug	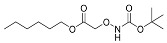	1%	0, 8, 21, 41%	63%	0%
**YD0242**	Prodrug	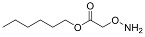	0%	0, 0, 2, 65%	0%	60%
**YD0246**	Prodrug	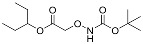	0%	0, 0, 0, 43%	54%	73%
**YD0251**	Prodrug	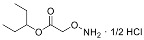	10%	5, 5, 11, 52%	42%	54%
**YD0335**	Prodrug	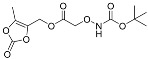	0%	0, 0, 16, 54%	46%	59%
**YD0343**	Prodrug	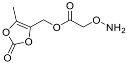	0%	0, 0, 0, 24%	0%	0%
**YD0381**	Prodrug	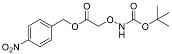	1%	0, 3, 13, 41%	81%	61%
**YD0382**	Prodrug (Hypoxia-activated)	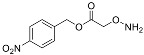	20%	0, 0, 0, 50%	0%	0%
**YD0450**	Prodrug	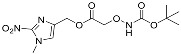	3%	0, 0, 0, 59%	16%	73%
**YD0452**	Prodrug (Hypoxia-activated)	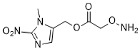	50%	0, 0, 0, 50%	0%	0%
**YD0541**	Prodrug (PEGylated)	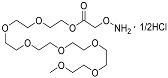	1%	0, 0, 0, 19%	0%	38%
**YD0706**	Prodrug	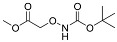	0%	0, 0, 0, 0%	0%	42%

## Data Availability

The data presented in this study are available on request from the corresponding authors.

## Supplementary Materials

The following are available online at https://www.mdpi.com/article/10.3390/biom11081073/s1.
