# Supplementary material for: Efficacy of Novel Aminooxyacetic Acid Prodrugs in Colon Cancer Models: Towards Clinical Translation of the Cystathionine β-Synthase Inhibition Concept
Source: Biomolecules. 2021 Jul 21;11(8):1073. doi: 10.3390/biom11081073 (PMC8394431; doi:10.3390/biom11081073)
Supplement: Supplementary file 1 [file biomolecules-11-01073-s001.zip › biomolecules-1300302-supplementary.pdf]

Supporting Information

Efficacy of novel aminooxyacetic acid prodrugs in colon cancer models: towards clinical translation of the cystathionine β-synthase inhibition concept

Mark R. Hellmich,[1*] Celia Chao,[1] Katalin Módis,[1,2] Ye Ding,[3] John R. Zatarain,[1] Ketan Thanki,[1] Manjit Maskey,[1] Nadiya Druzhyna,[2] Ashley A. Untereiner,[2] Akbar Ahmad,[2] Yu Xue,[3] Haiying Chen,[3] William K. Russell,[4] Jianmei Wang,[5] Jia Zhou,[3*] and Csaba Szabo[2,6*]

[1]Department of Surgery, [2]Department of Anesthesiology, [3]Department of Pharmacology and Toxicology, and [4]Department of Biochemistry and Molecular Biology, University of Texas, Medical Branch, Galveston, Texas, United States of America, [5]College of Pharmacy, University of North Texas Health Science Center, Fort Worth, Texas, United States of America and [6]Chair of Pharmacology, Section of Medicine, University of Fribourg, CH-1700 Fribourg, Switzerland.

Copies of ^{1}H NMR and ^{13}C NMR Spectra of New Molecules

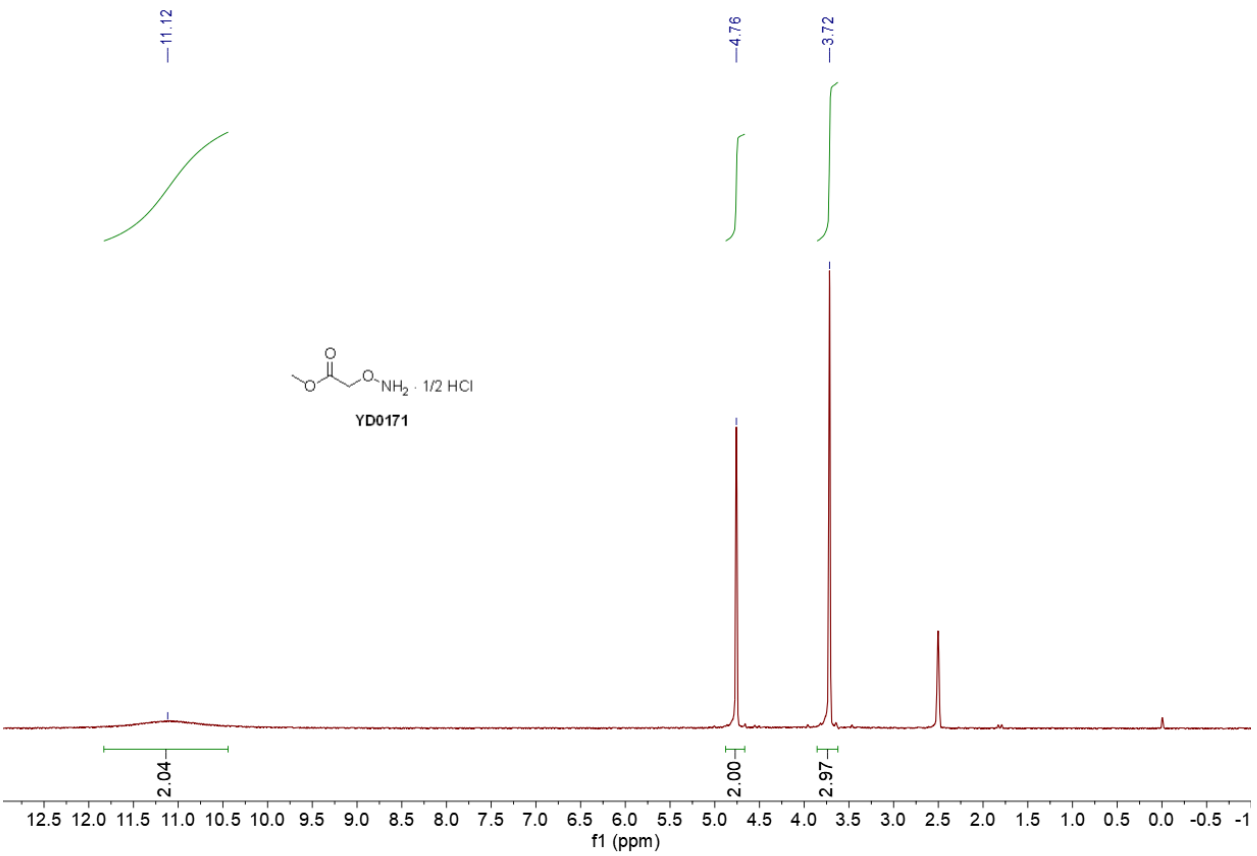

11.12
4.76
3.72
1/2 HCl
YD0171
2.04
2.00
2.97
12.5 12.0 11.5 11.0 10.5 10.0 9.5 9.0 8.5 8.0 7.5 7.0 6.5 6.0 5.5 5.0 4.5 4.0 3.5 3.0 2.5 2.0 1.5 1.0 0.5 0.0 -0.5 -1
f1 (ppm)

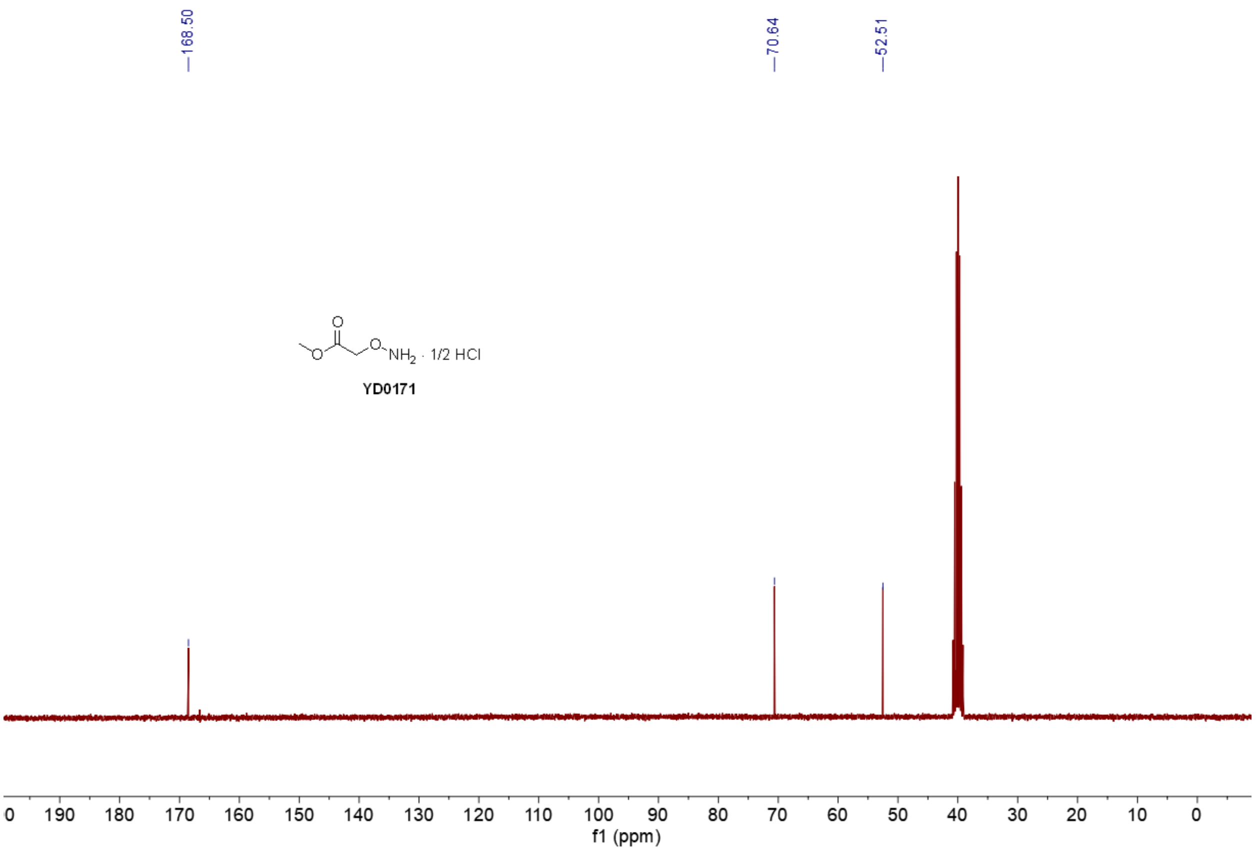

168.50
70.64
52.51
1/2 HCl
YD0171
0 190 180 170 160 150 140 130 120 110 100 90 80 70 60 50 40 30 20 10 0
f1 (ppm)

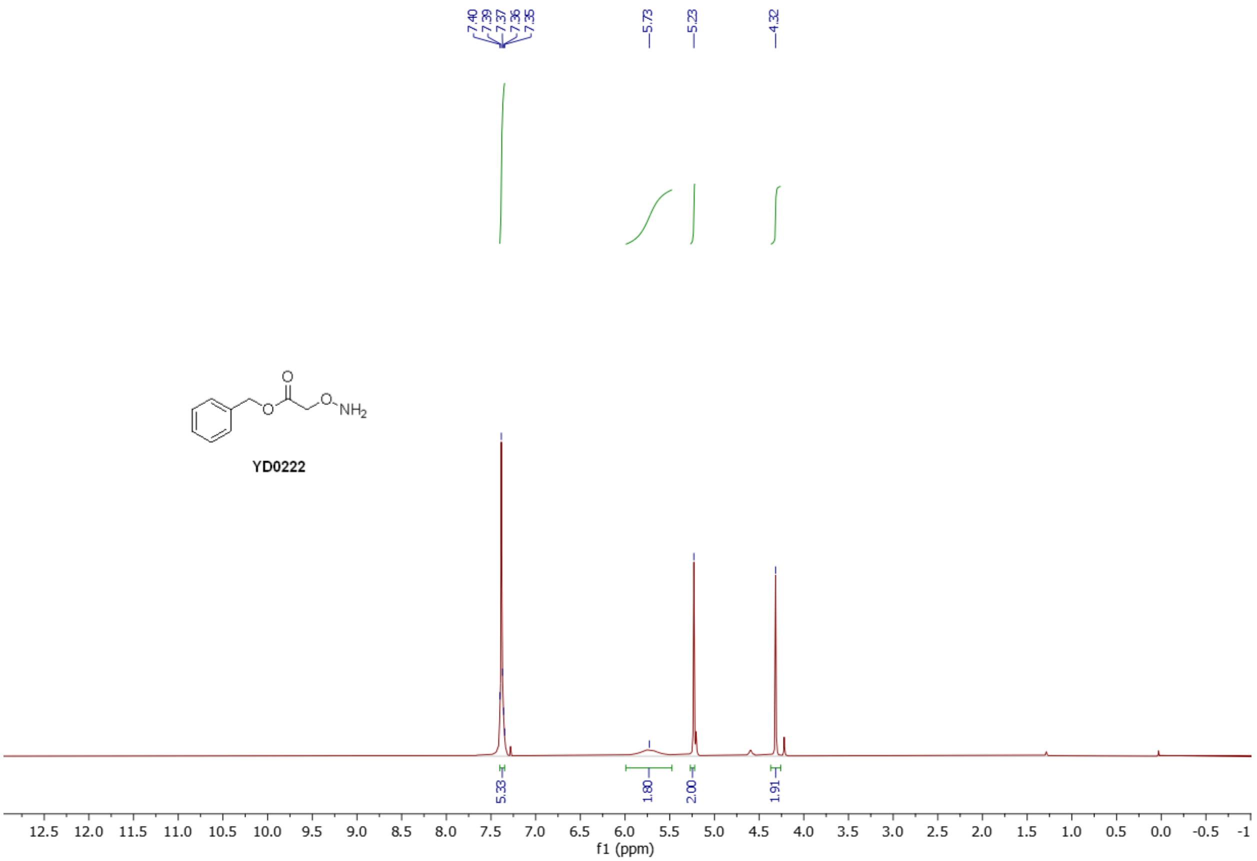
7.40
7.39
7.37
7.36
7.35
5.73
5.23
4.32
NH2
YD0222
5.33
1.80
2.00
1.91
12.5 12.0 11.5 11.0 10.5 10.0 9.5 9.0 8.5 8.0 7.5 7.0 6.5 6.0 5.5 5.0 4.5 4.0 3.5 3.0 2.5 2.0 1.5 1.0 0.5 0.0 -0.5 -1
f1 (ppm)

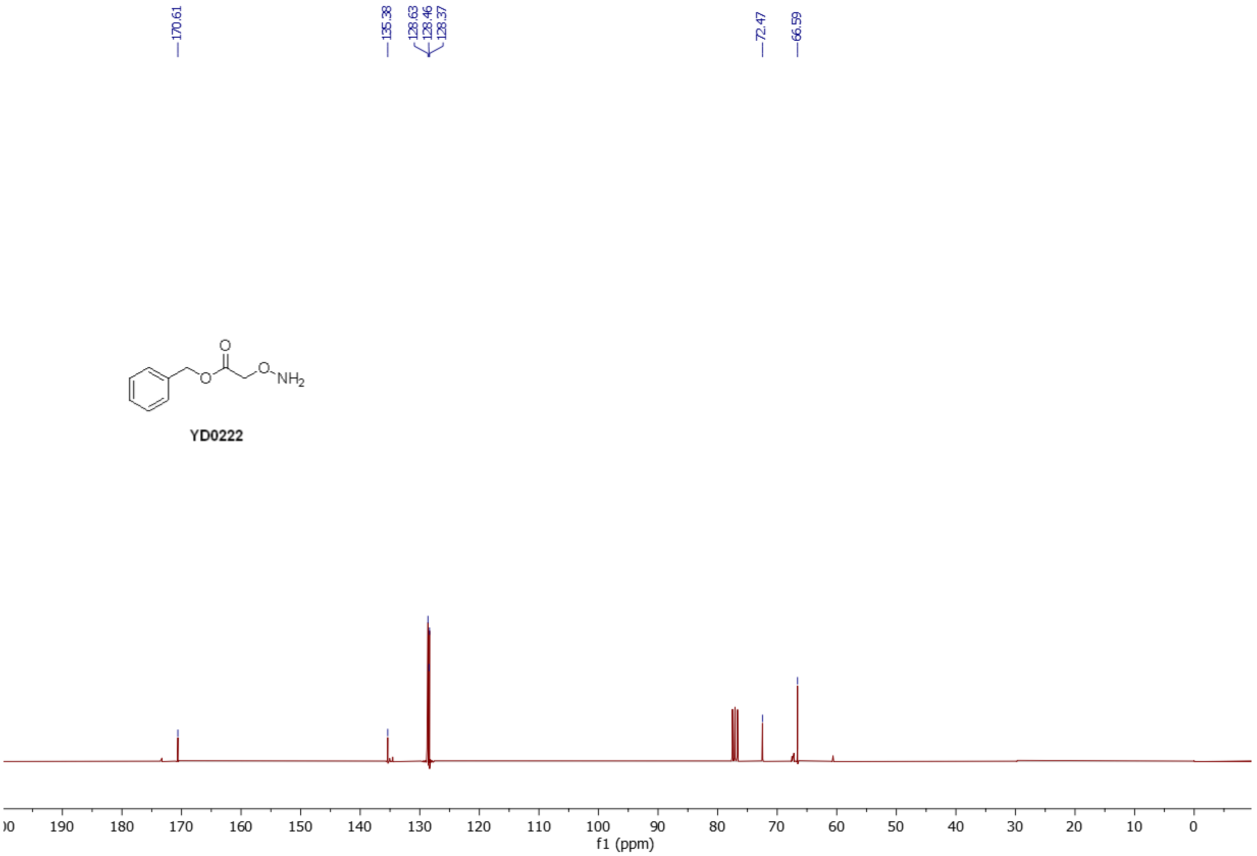
170.61
135.38
128.63
128.46
128.37
72.47
66.59
NH2
YD0222
190 180 170 160 150 140 130 120 110 100 90 80 70 60 50 40 30 20 10 0
f1 (ppm)

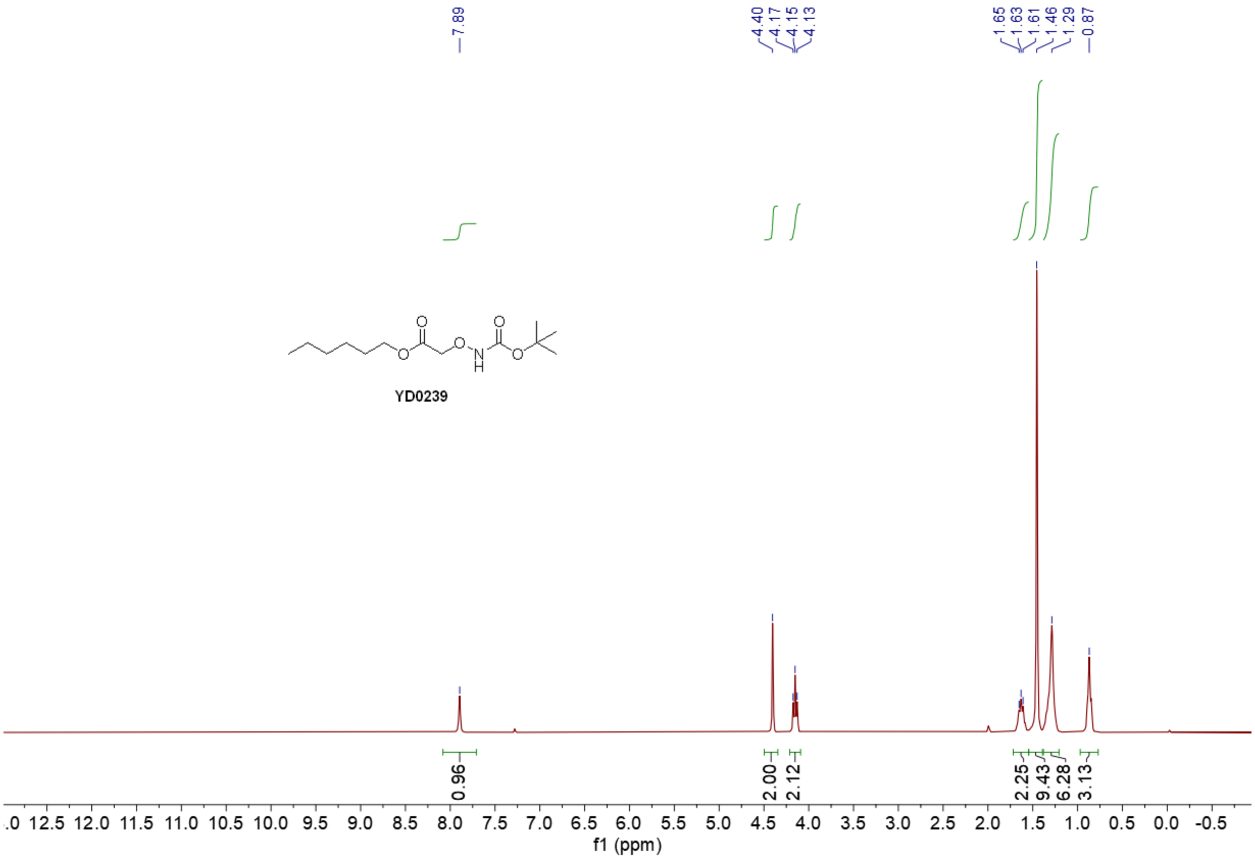

7.89
4.40
4.17
4.15
4.13
1.65
1.63
1.61
1.46
1.29
0.87
YD0239
0.96
2.00
2.12
2.25
9.43
6.28
3.13
f1 (ppm)

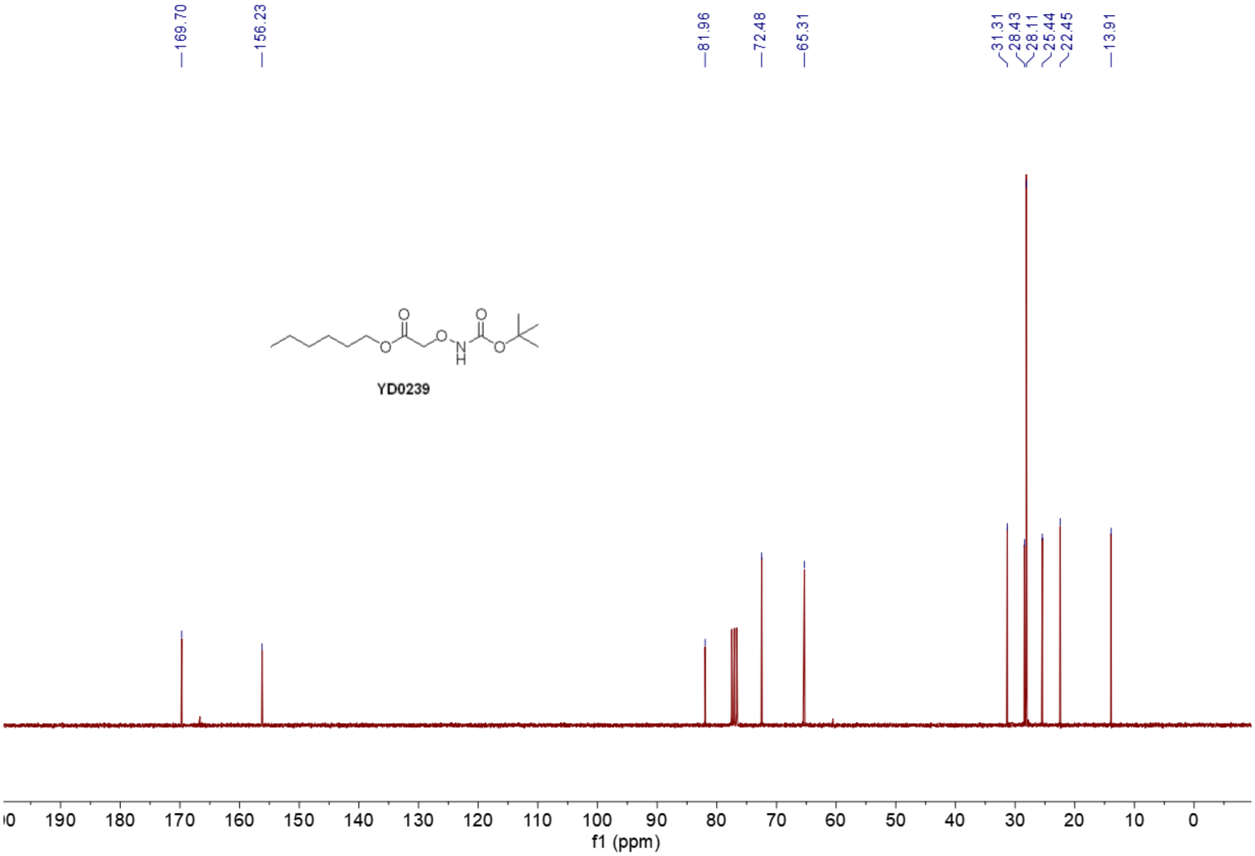

169.70
156.23
81.96
72.48
65.31
31.31
28.43
28.11
25.44
22.45
13.91
YD0239
f1 (ppm)

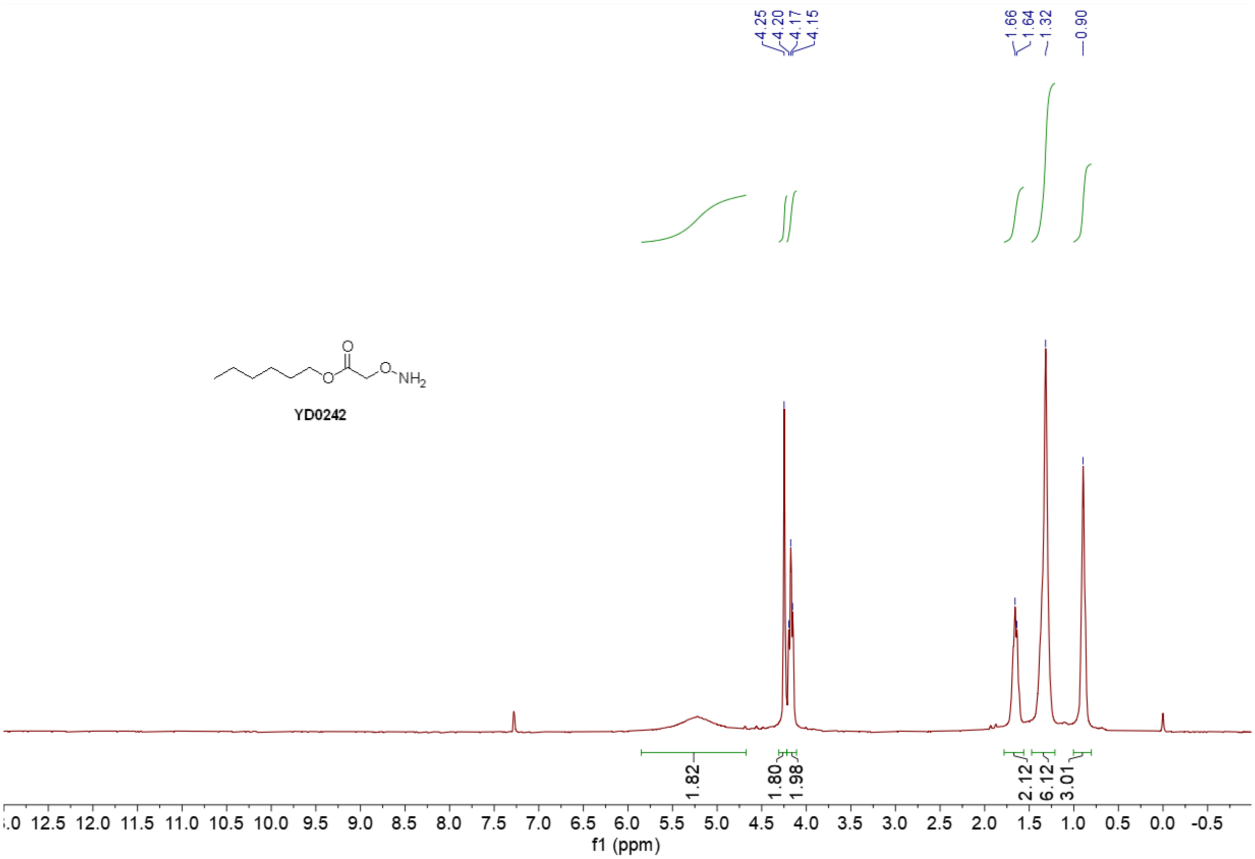
4.25
4.20
4.17
4.15
1.66
1.64
1.32
0.90
YD0242
1.82
1.80
1.98
2.12
6.12
3.01
f1 (ppm)

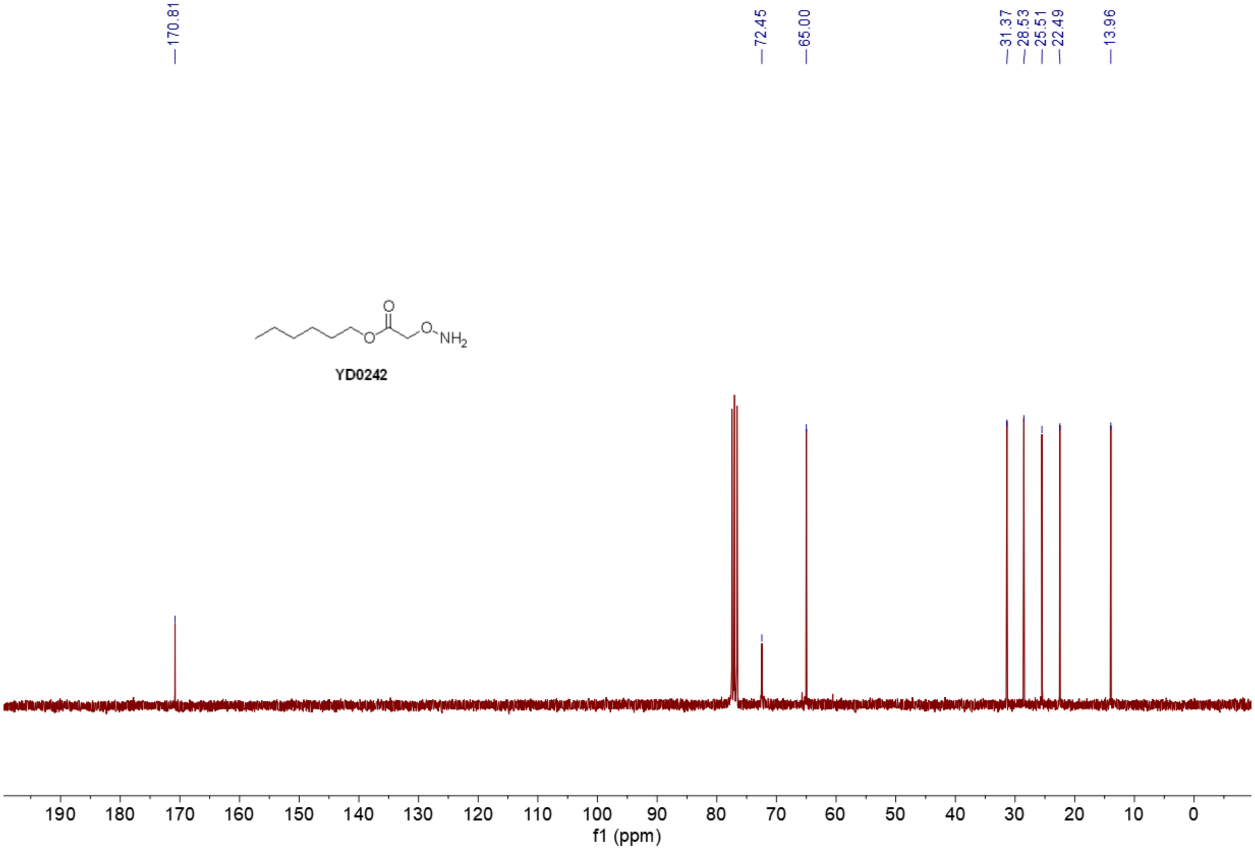
170.81
72.45
65.00
31.37
28.53
25.51
22.49
13.96
YD0242
f1 (ppm)

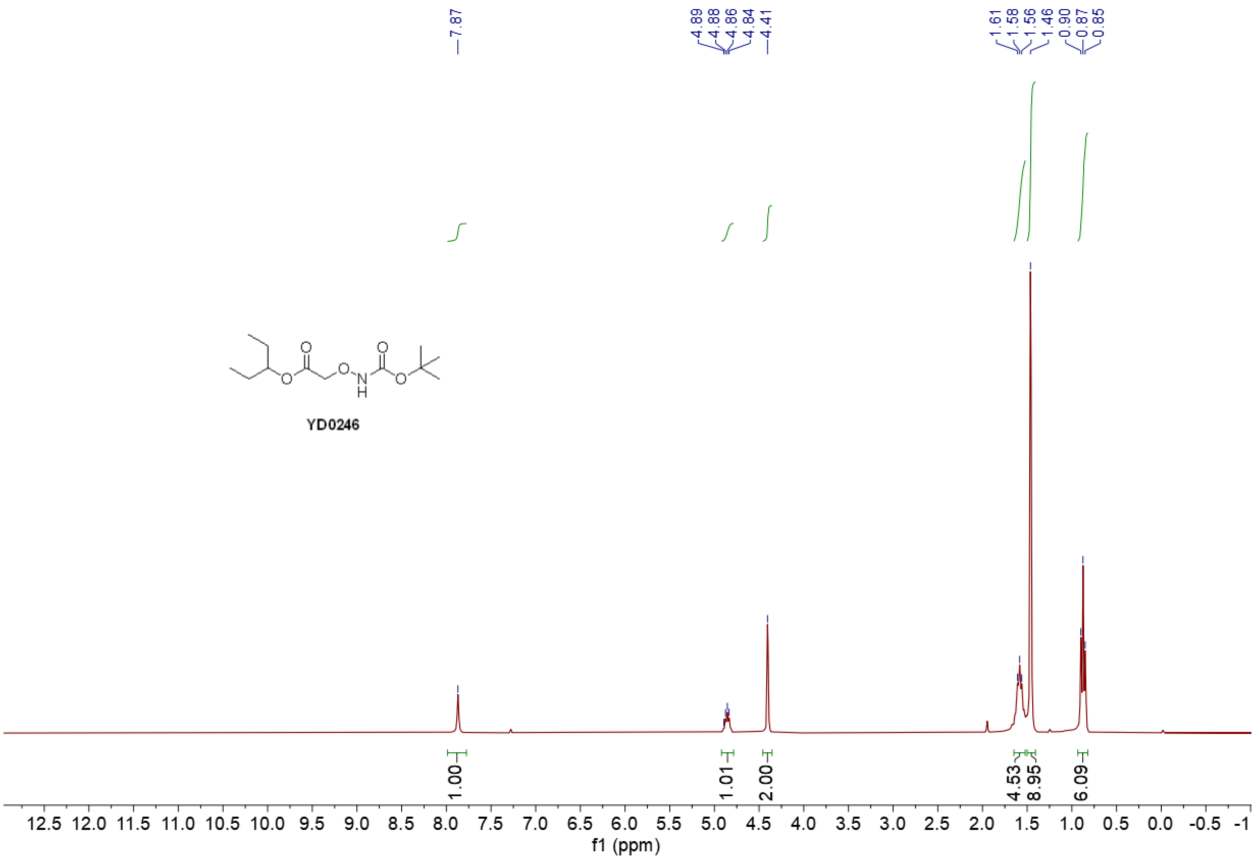
7.87
4.89 4.88 4.86 4.84 4.41
1.61 1.58 1.56 1.46 0.90 0.87 0.85
YD0246
1.00 1.01 2.00 4.53 8.95 6.09
f1 (ppm)

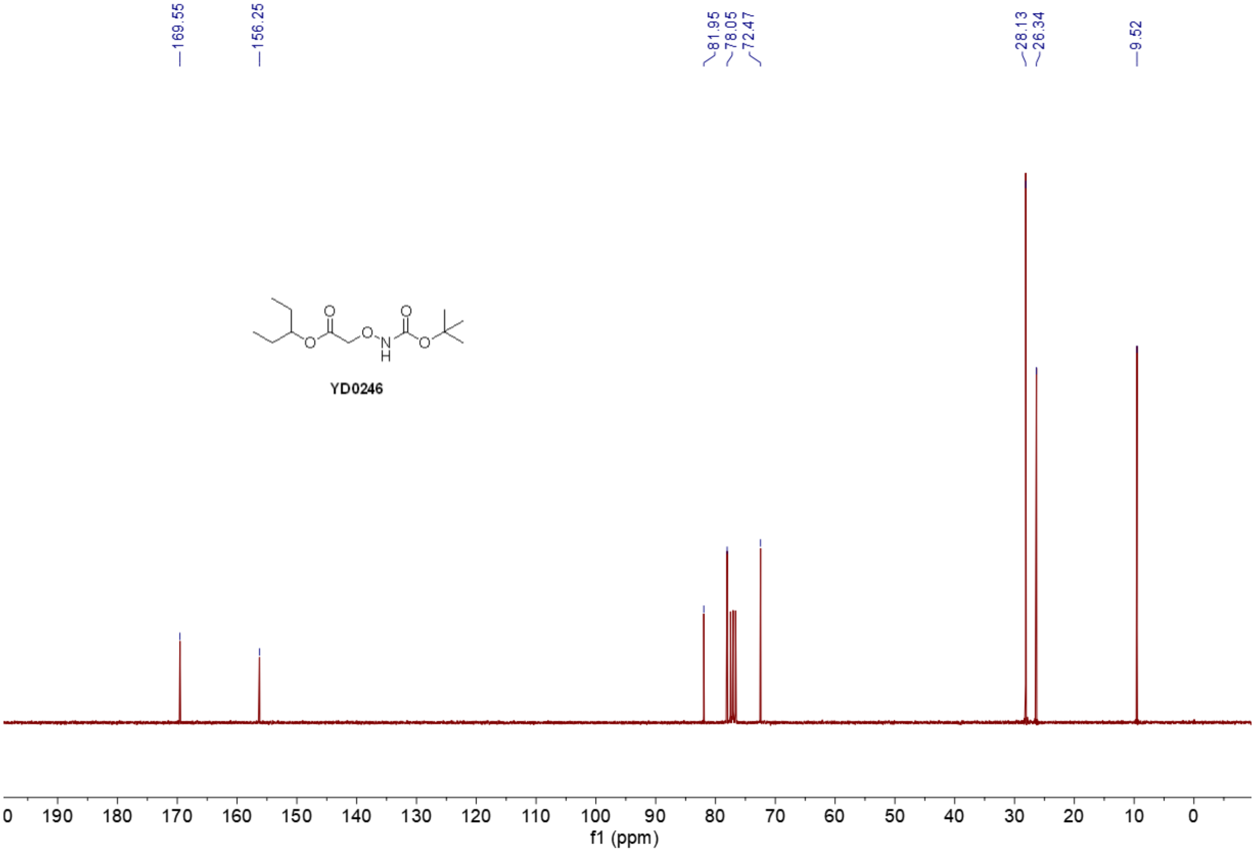
169.55
156.25
81.95 78.05 72.47
28.13 26.34
9.52
YD0246
f1 (ppm)

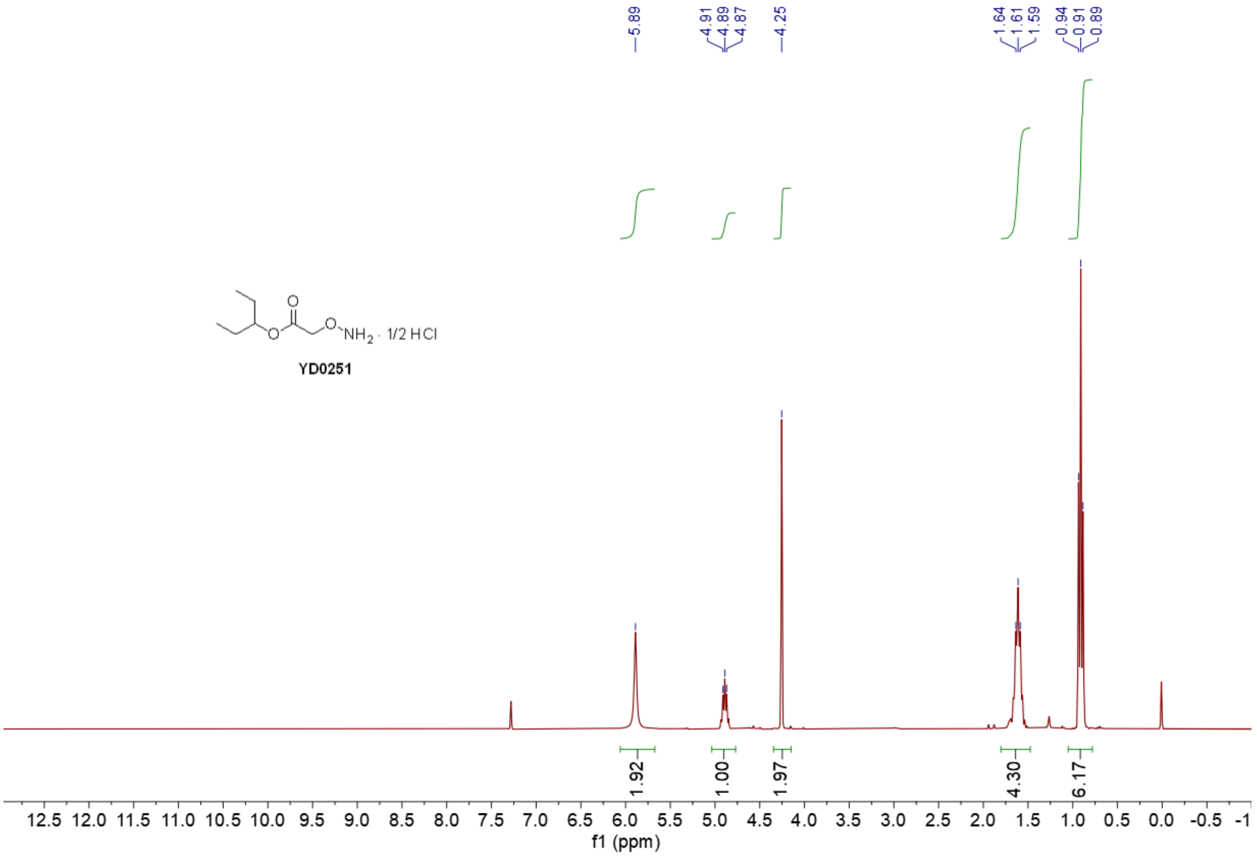
5.89
4.91
4.89
4.87
4.25
1.64
1.61
1.59
0.94
0.91
0.89
NH2 · 1/2 HCl
YD0251
1.92
1.00
1.97
4.30
6.17
12.5 12.0 11.5 11.0 10.5 10.0 9.5 9.0 8.5 8.0 7.5 7.0 6.5 6.0 5.5 5.0 4.5 4.0 3.5 3.0 2.5 2.0 1.5 1.0 0.5 0.0 -0.5 -1
f1 (ppm)

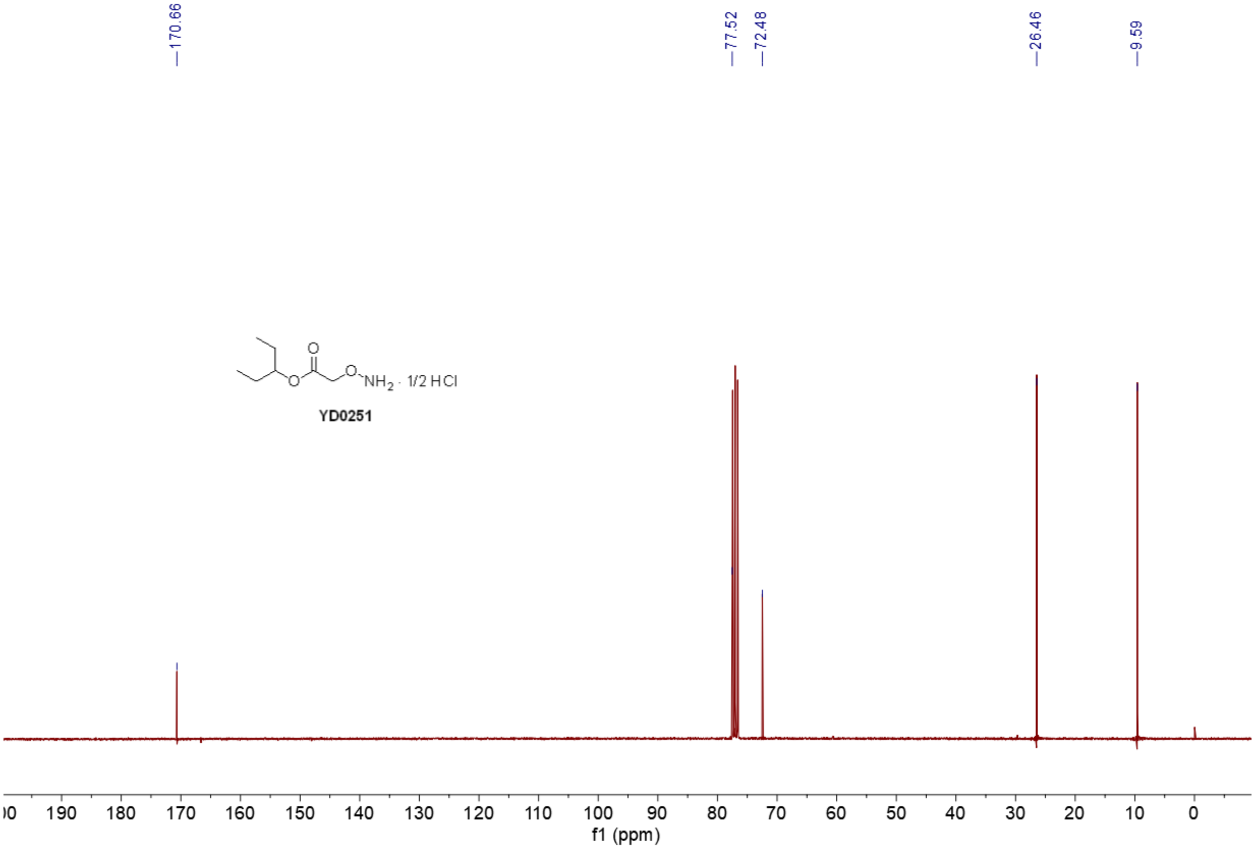
170.66
77.52
72.48
26.46
9.59
NH2 · 1/2 HCl
YD0251
190 180 170 160 150 140 130 120 110 100 90 80 70 60 50 40 30 20 10 0
f1 (ppm)

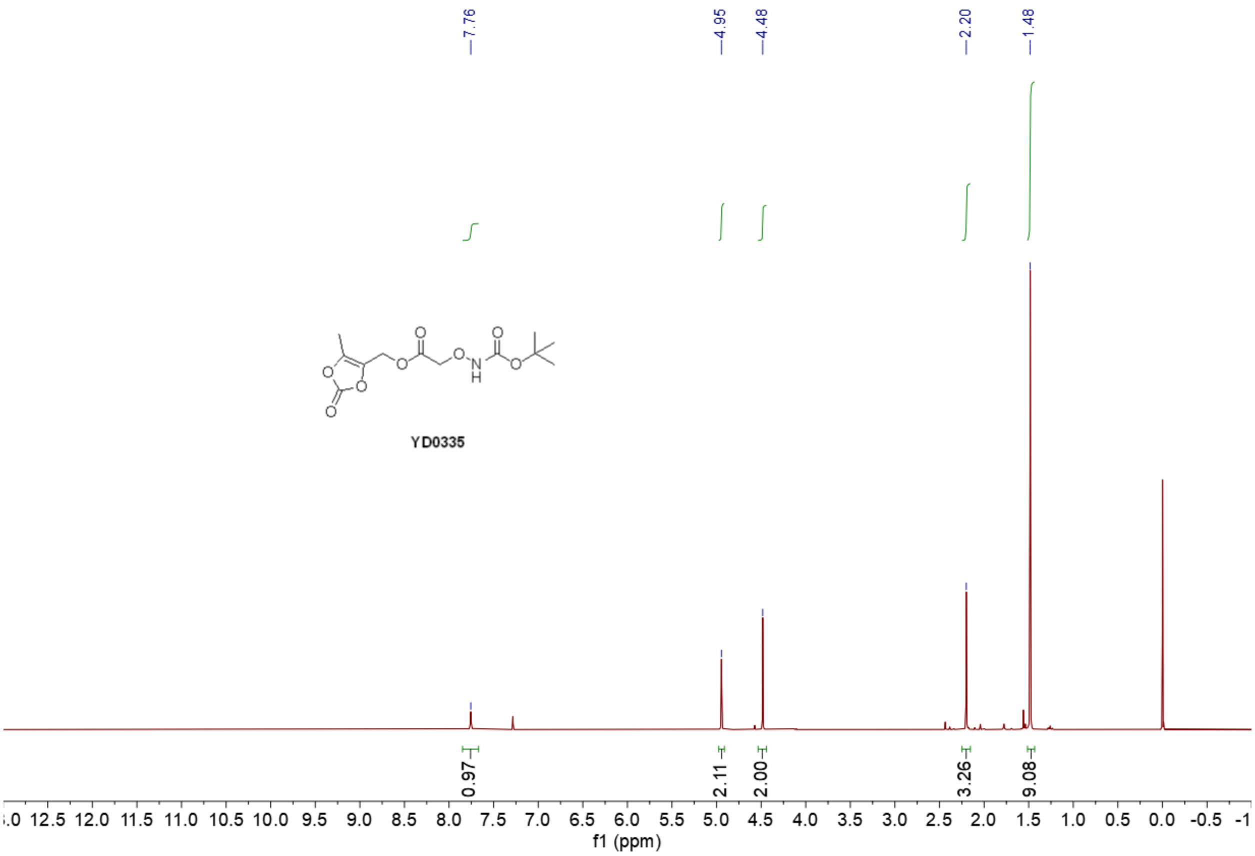
7.76
4.95
4.48
2.20
1.48
YD0335
0.97
2.11
2.00
3.26
9.08
f1 (ppm)

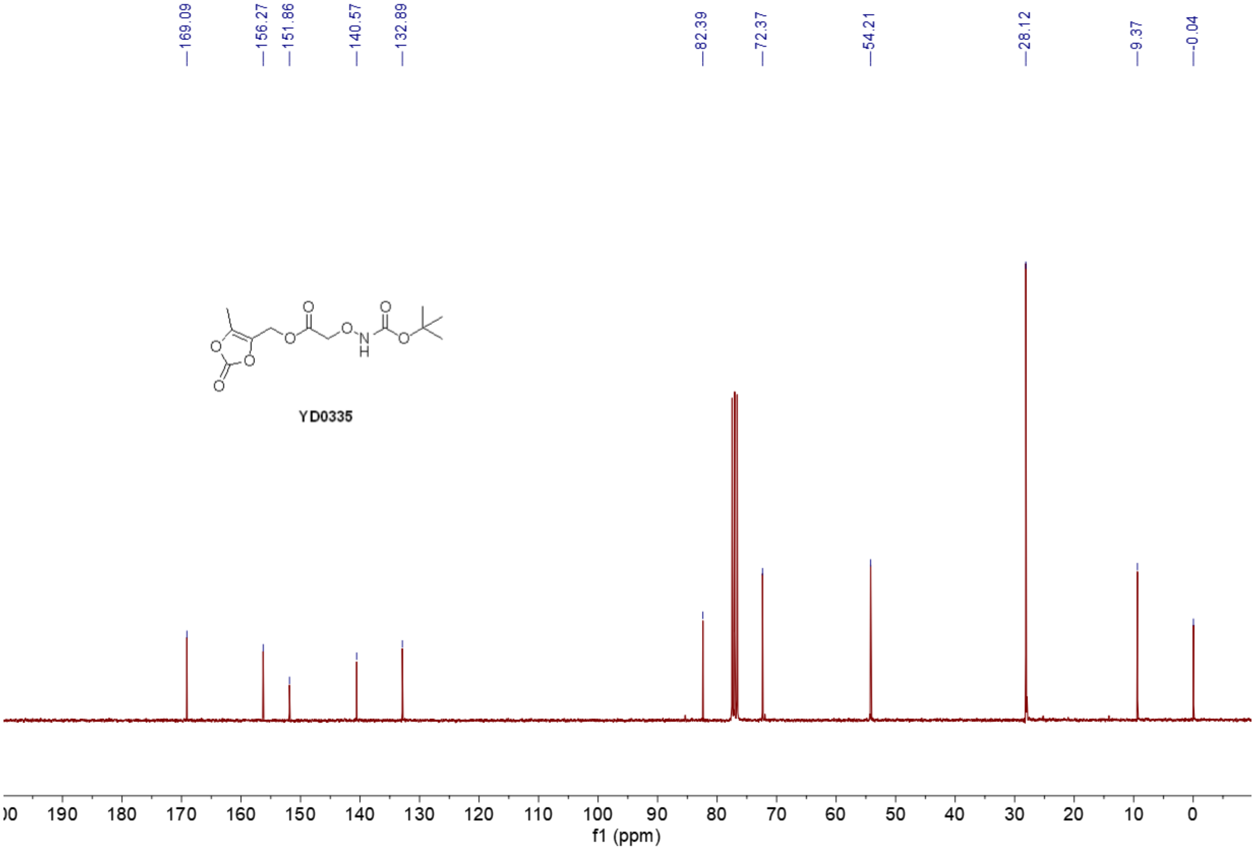
169.09
156.27
151.86
140.57
132.89
82.39
72.37
54.21
28.12
9.37
-0.04
YD0335
f1 (ppm)

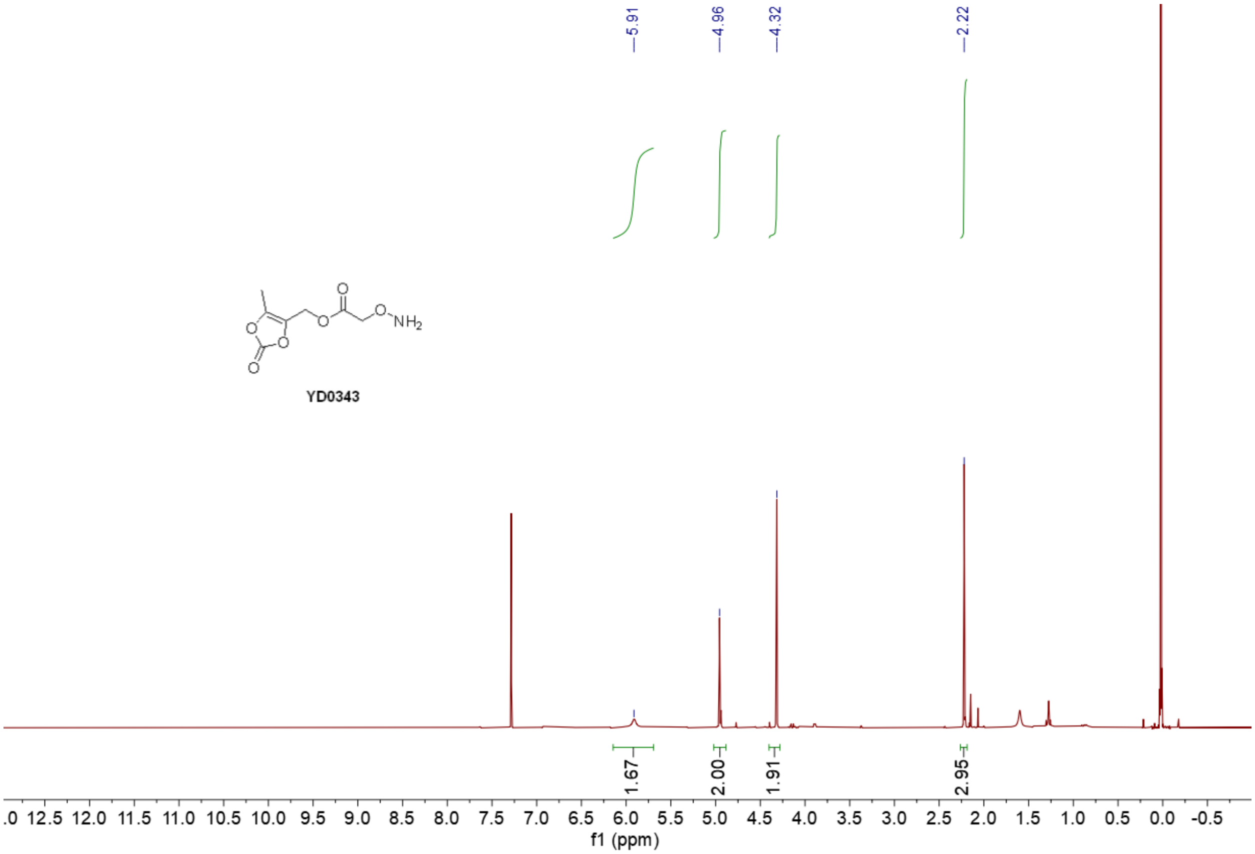
5.91
4.96
4.32
2.22
YD0343
1.67
2.00
1.91
2.95
13.0 12.5 12.0 11.5 11.0 10.5 10.0 9.5 9.0 8.5 8.0 7.5 7.0 6.5 6.0 5.5 5.0 4.5 4.0 3.5 3.0 2.5 2.0 1.5 1.0 0.5 0.0 -0.5
f1 (ppm)

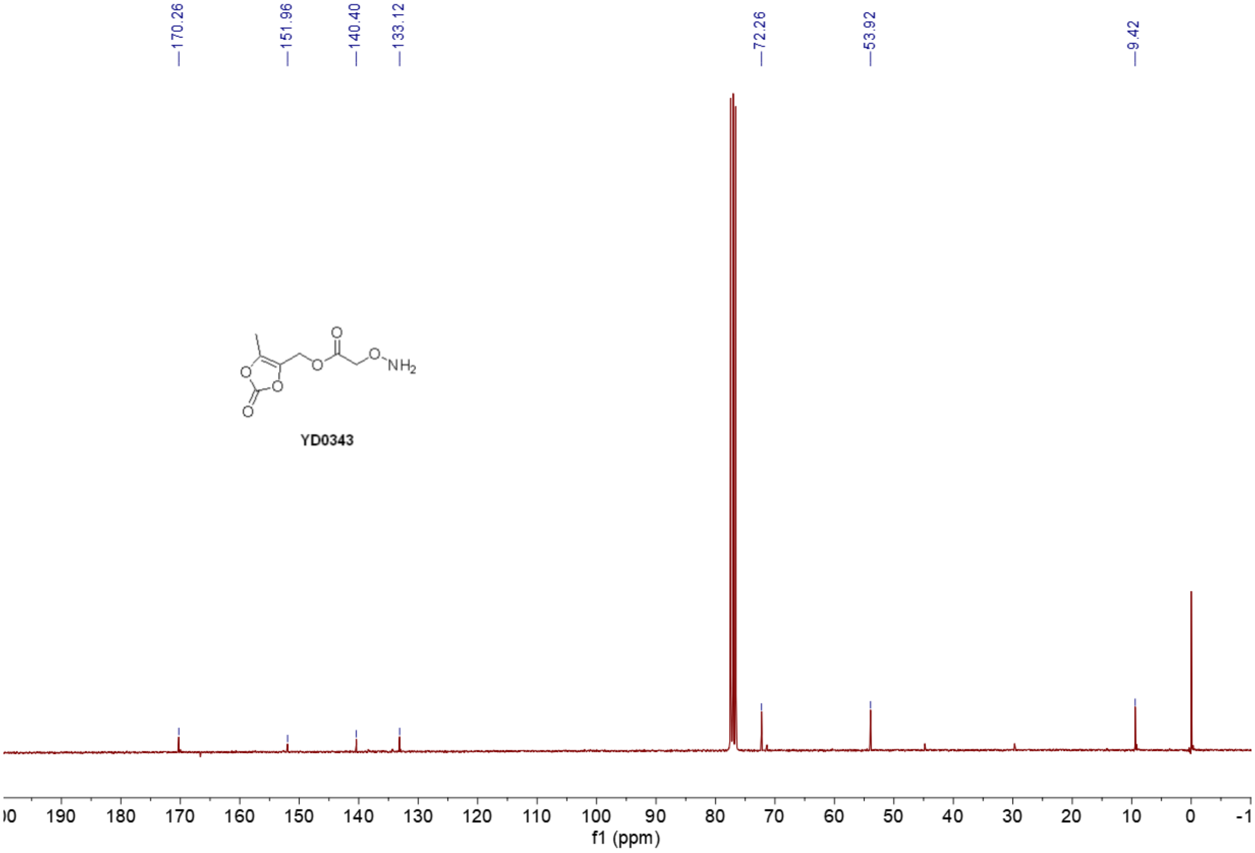
170.26
151.96
140.40
133.12
72.26
53.92
9.42
YD0343
190 180 170 160 150 140 130 120 110 100 90 80 70 60 50 40 30 20 10 0 -1
f1 (ppm)

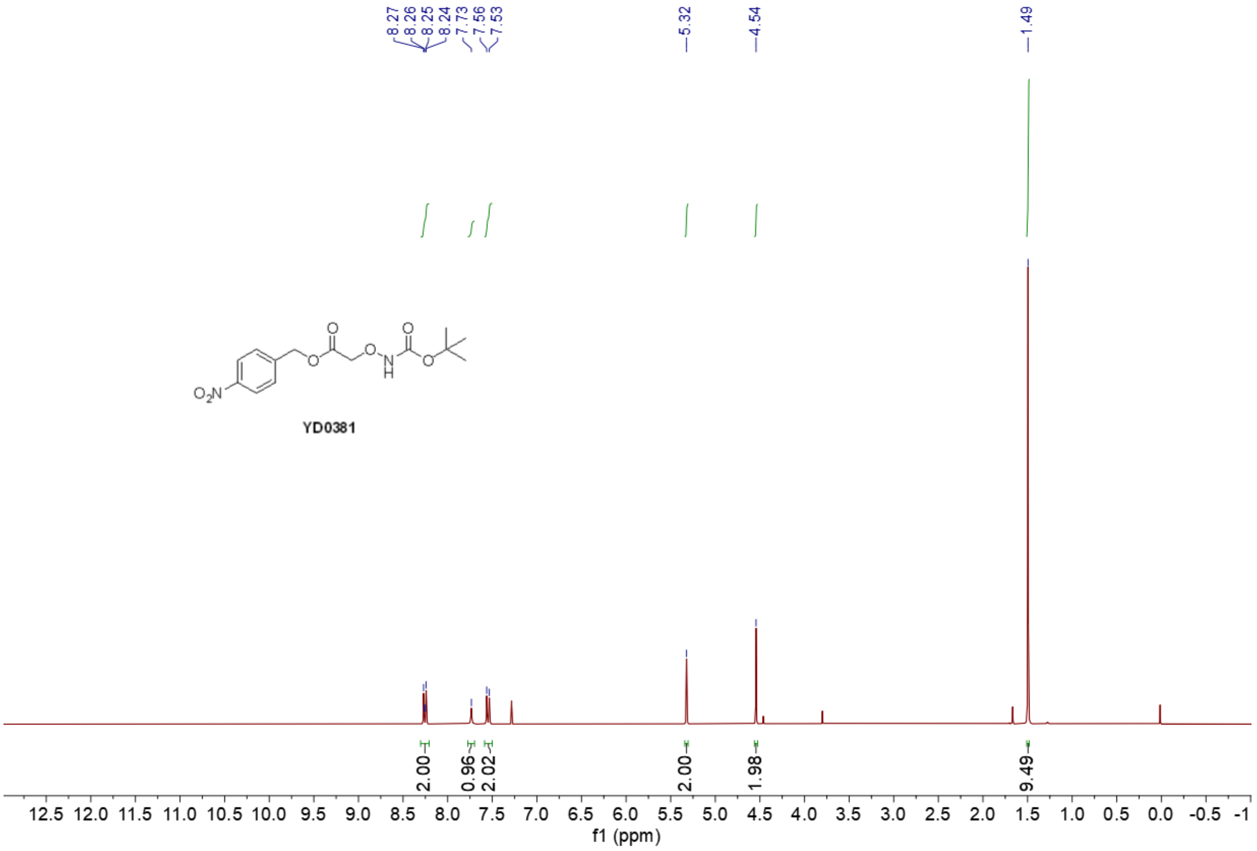
8.27
8.26
8.25
8.24
7.73
7.56
7.53
5.32
4.54
1.49
O2N
O
O
O
N
H
O
O
O
YD0381
2.00
0.96
2.02
2.00
1.98
9.49
12.5 12.0 11.5 11.0 10.5 10.0 9.5 9.0 8.5 8.0 7.5 7.0 6.5 6.0 5.5 5.0 4.5 4.0 3.5 3.0 2.5 2.0 1.5 1.0 0.5 0.0 -0.5 -1
f1 (ppm)

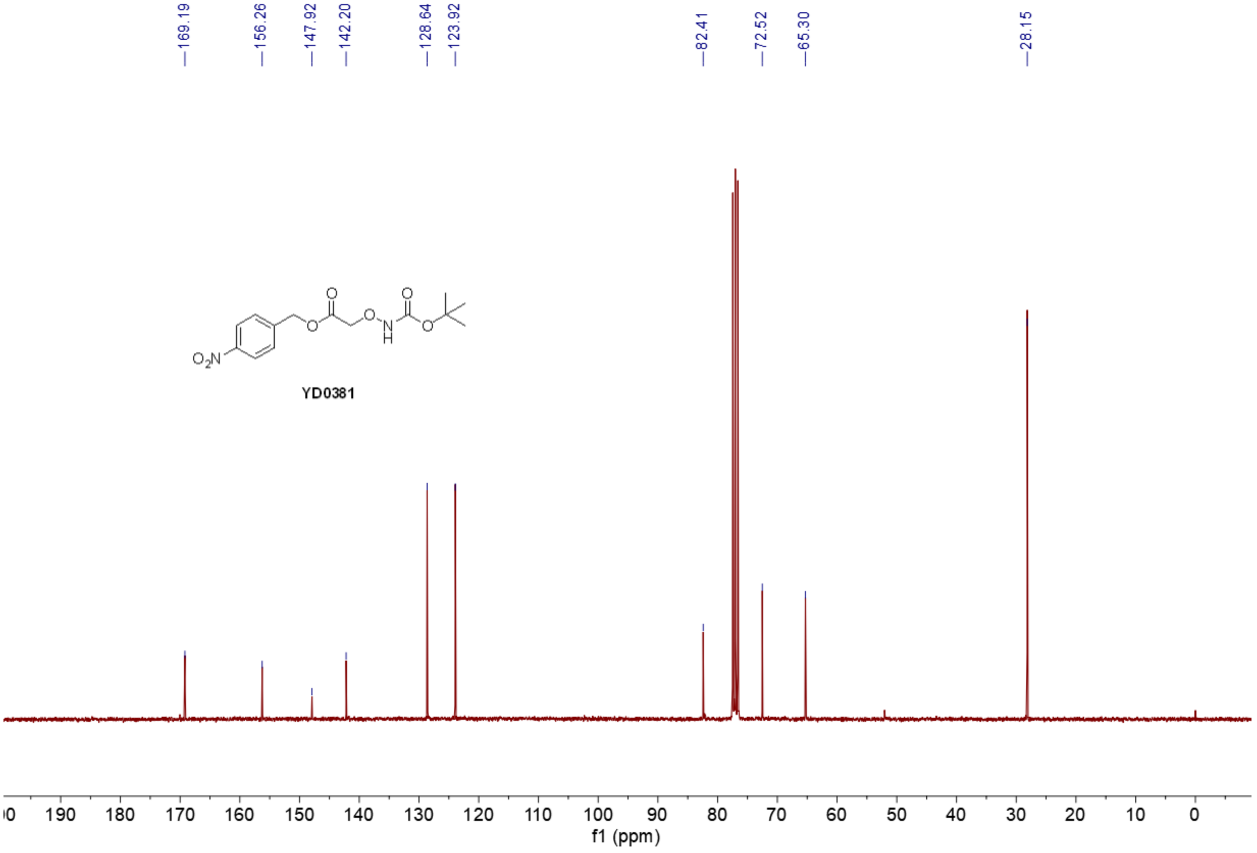
169.19
156.26
147.92
142.20
128.64
123.92
82.41
72.52
65.30
28.15
O2N
YD0381
190 180 170 160 150 140 130 120 110 100 90 80 70 60 50 40 30 20 10 0
f1 (ppm)

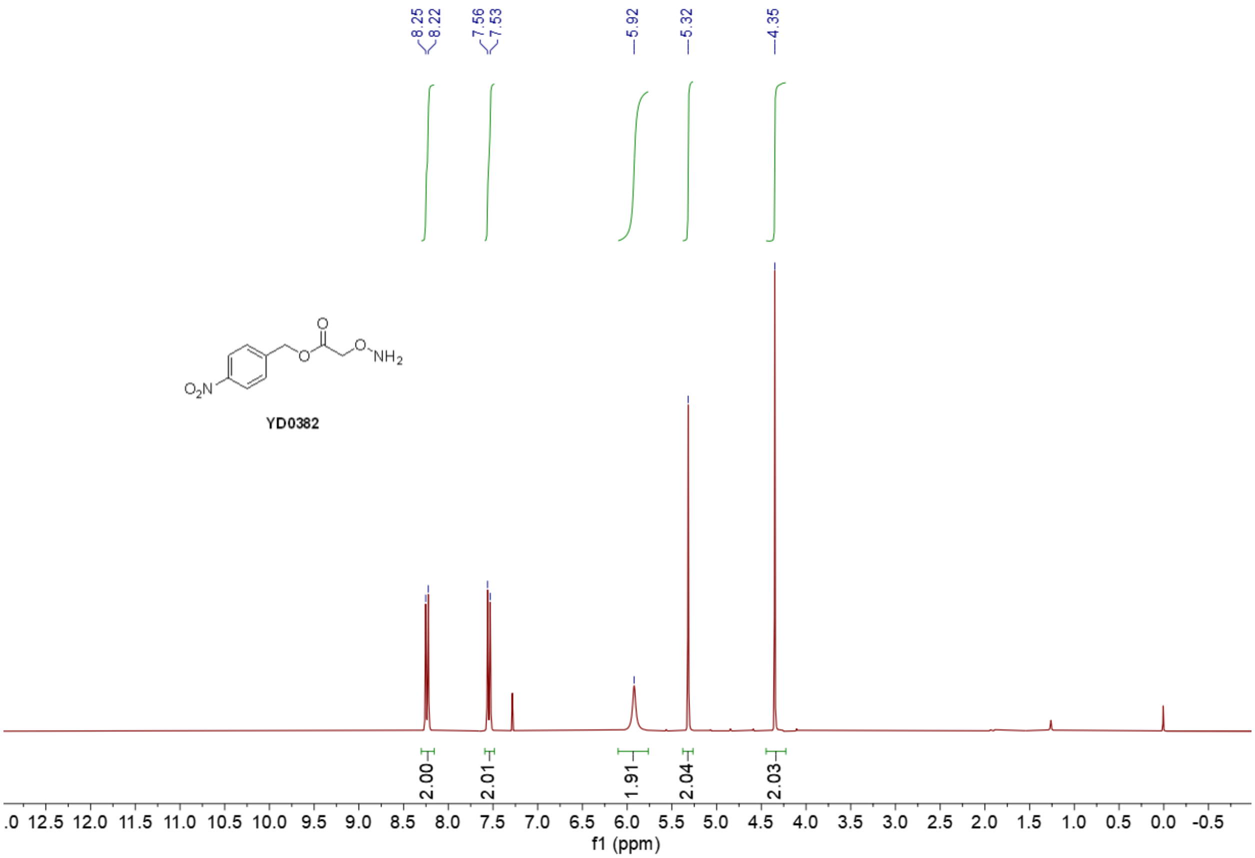

8.25
8.22
7.56
7.53
5.92
5.32
4.35
2.00
2.01
1.91
2.04
2.03
O
O
O
NH2
O2N
YD0382
f1 (ppm)

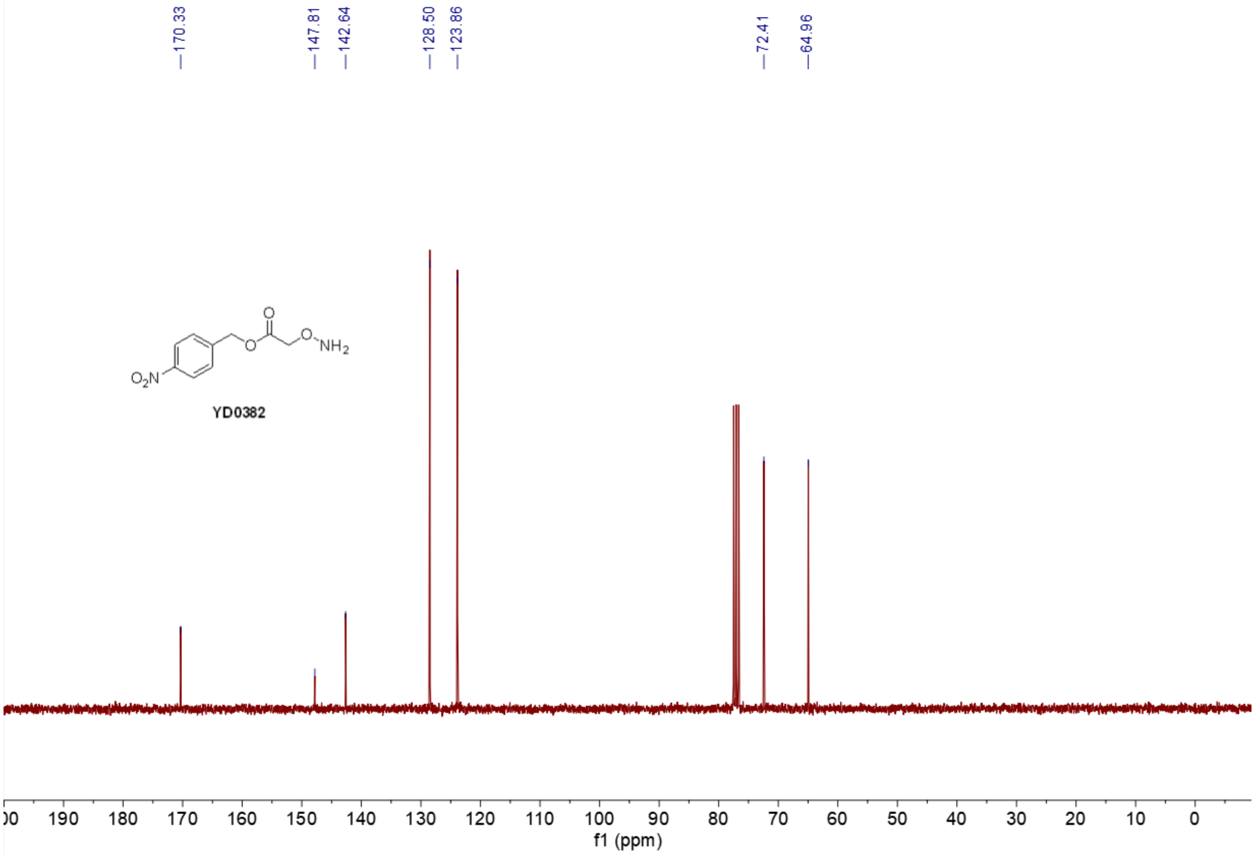

170.33
147.81
142.64
128.50
123.86
72.41
64.96
O
O
O
NH2
O2N
YD0382
f1 (ppm)

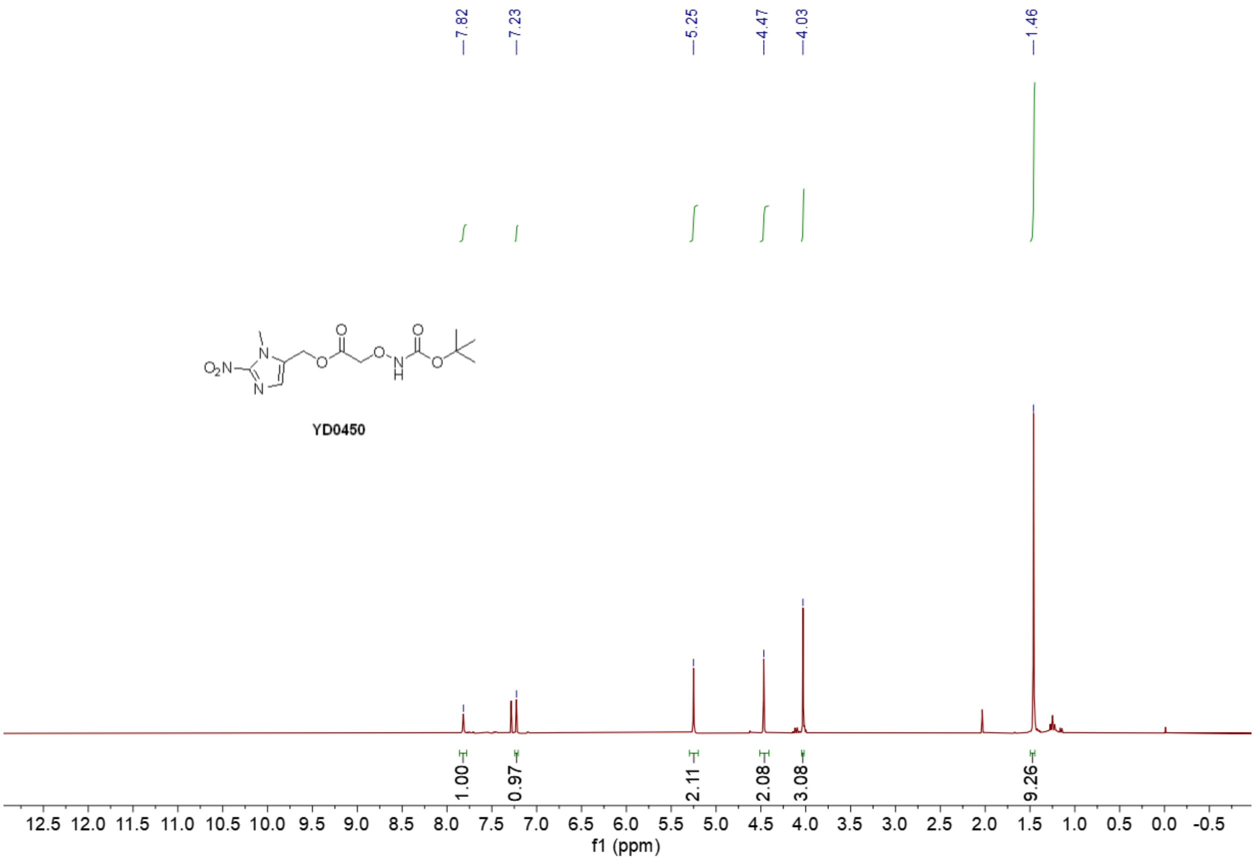
7.82
7.23
5.25
4.47
4.03
1.46
O_2N
N
N
O
O
O
N
H
O
O
YD0450
1.00
0.97
2.11
2.08
3.08
9.26
12.5 12.0 11.5 11.0 10.5 10.0 9.5 9.0 8.5 8.0 7.5 7.0 6.5 6.0 5.5 5.0 4.5 4.0 3.5 3.0 2.5 2.0 1.5 1.0 0.5 0.0 -0.5
f1 (ppm)

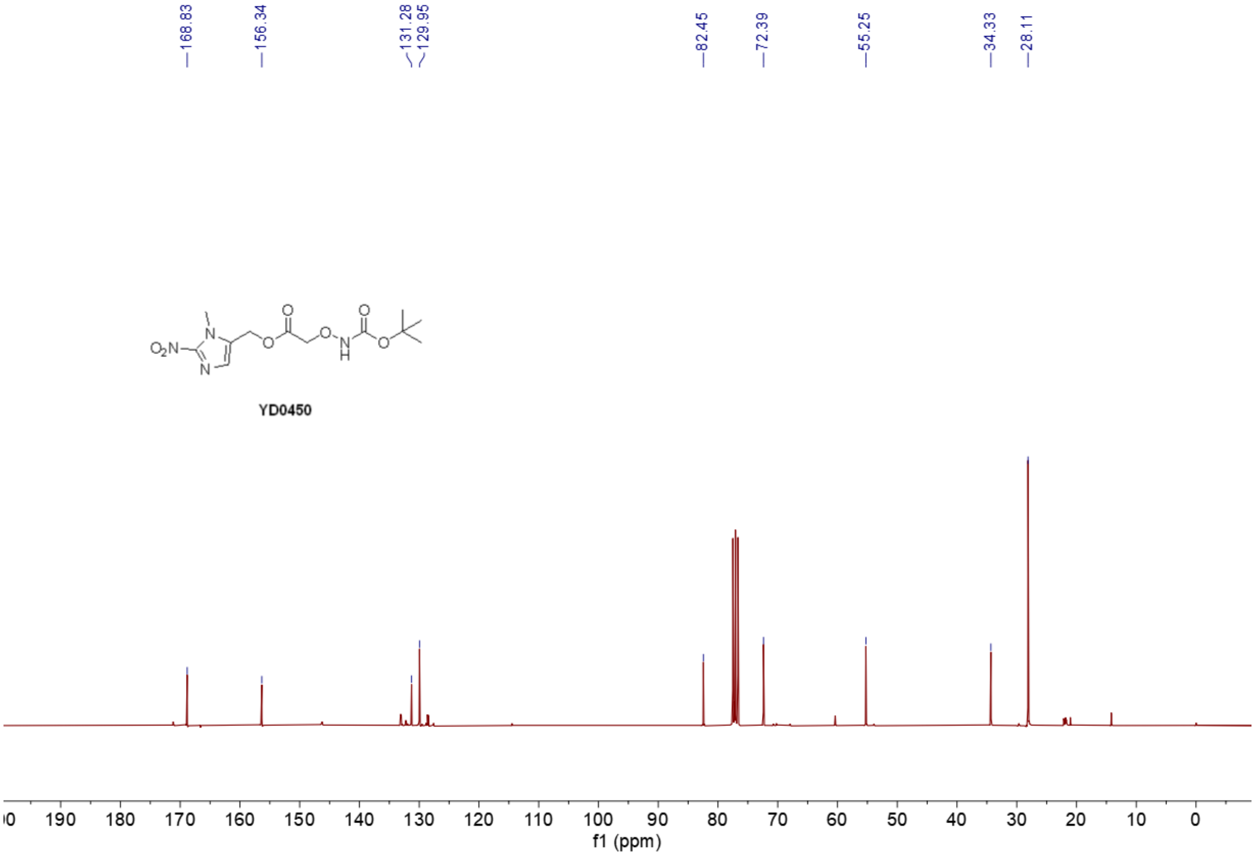
168.83
156.34
131.28
129.95
82.45
72.39
55.25
34.33
28.11
O_2N
N
N
O
O
O
N
H
O
O
YD0450
190 180 170 160 150 140 130 120 110 100 90 80 70 60 50 40 30 20 10 0
f1 (ppm)

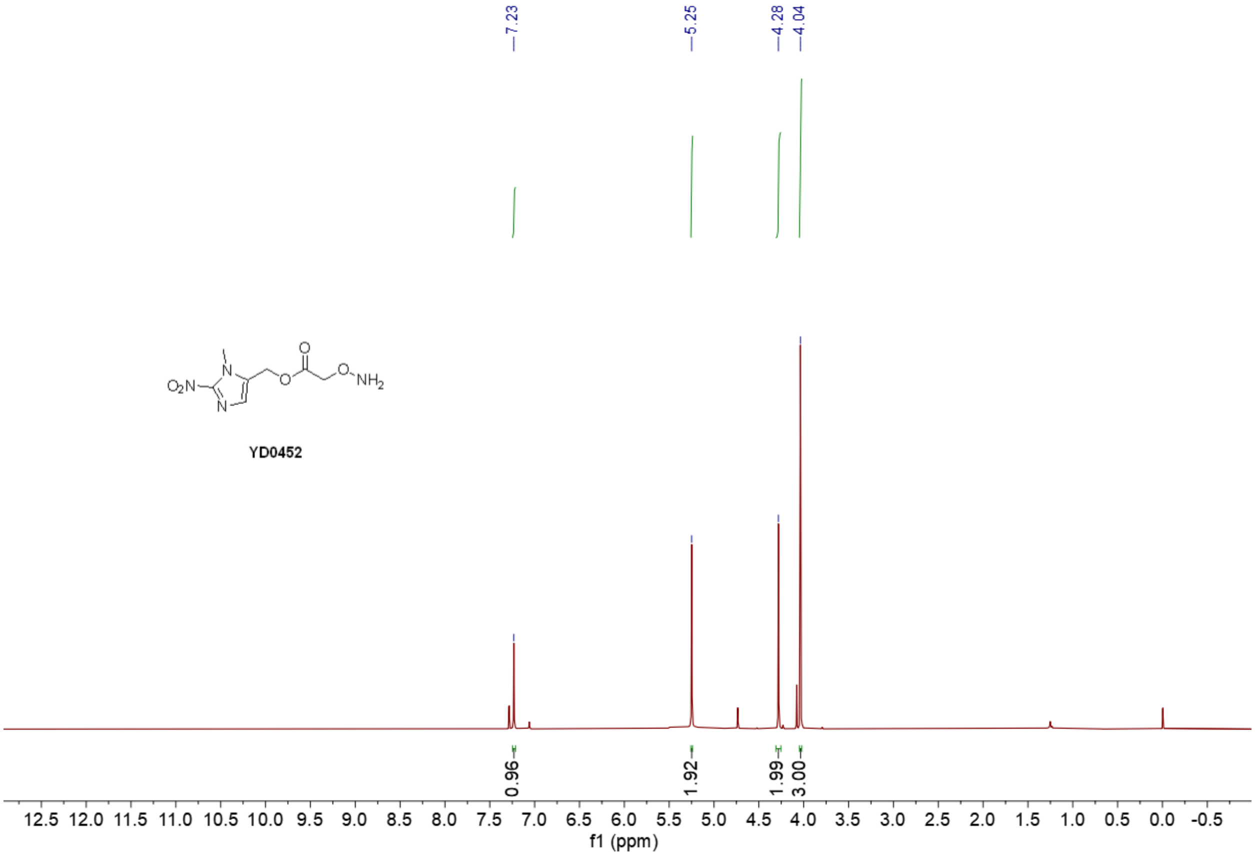
7.23
5.25
4.28
4.04
O_2N
N
O
O
NH_2
YD0452
0.96
1.92
1.99
3.00
12.5 12.0 11.5 11.0 10.5 10.0 9.5 9.0 8.5 8.0 7.5 7.0 6.5 6.0 5.5 5.0 4.5 4.0 3.5 3.0 2.5 2.0 1.5 1.0 0.5 0.0 -0.5
f1 (ppm)

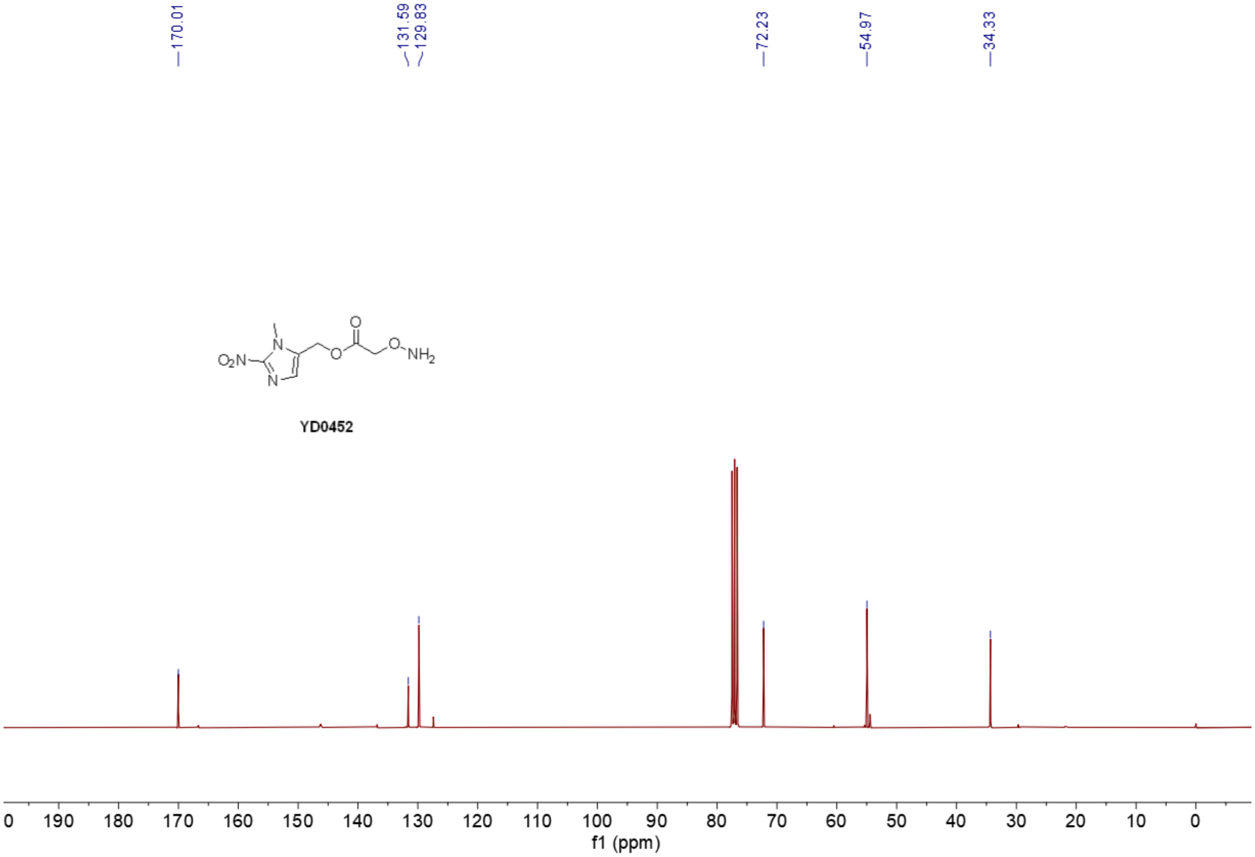
170.01
131.59
129.83
72.23
54.97
34.33
O_2N
N
O
O
NH_2
YD0452
0 190 180 170 160 150 140 130 120 110 100 90 80 70 60 50 40 30 20 10 0
f1 (ppm)

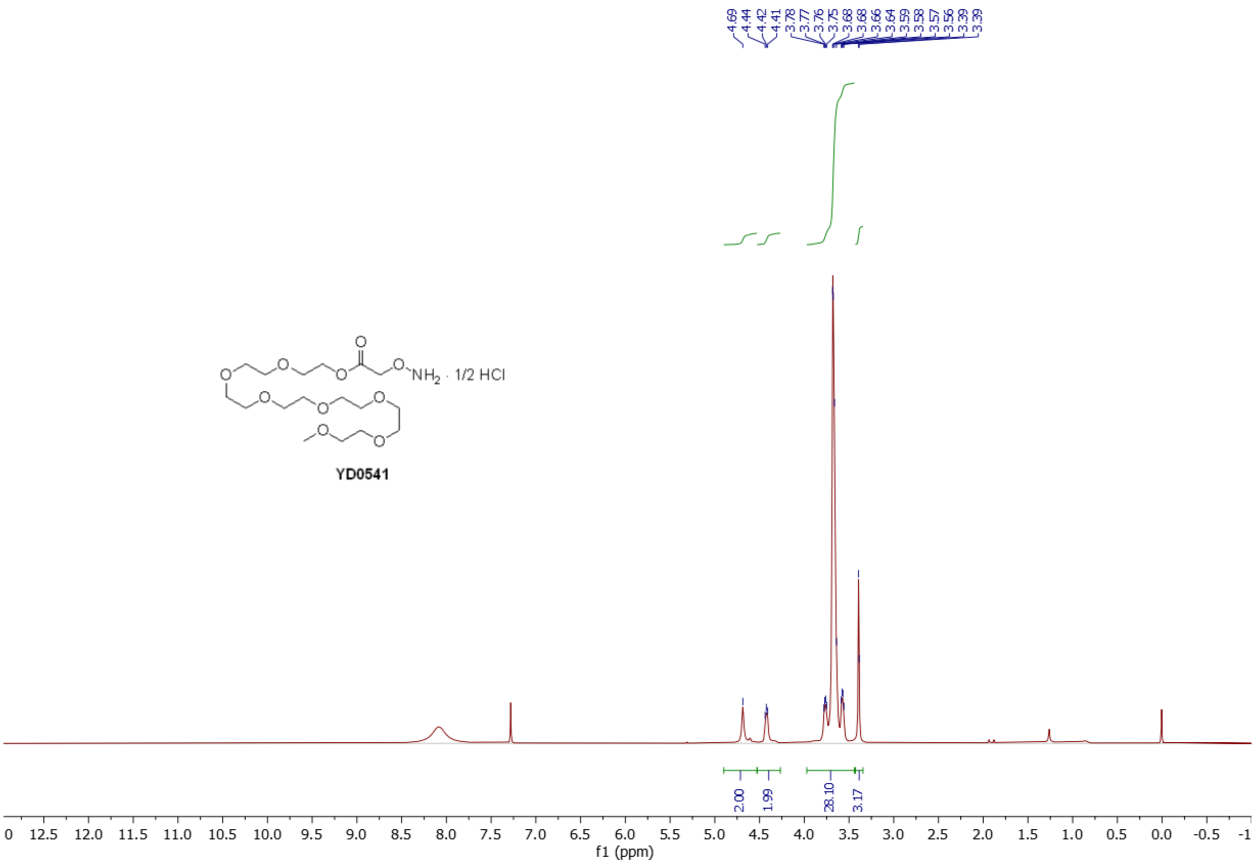

4.69
4.44
4.42
4.41
3.78
3.77
3.76
3.75
3.68
3.68
3.66
3.64
3.59
3.58
3.57
3.56
3.39
3.39
2.00
1.99
28.10
3.17
f1 (ppm)
NH2 · 1/2 HCl
YD0541

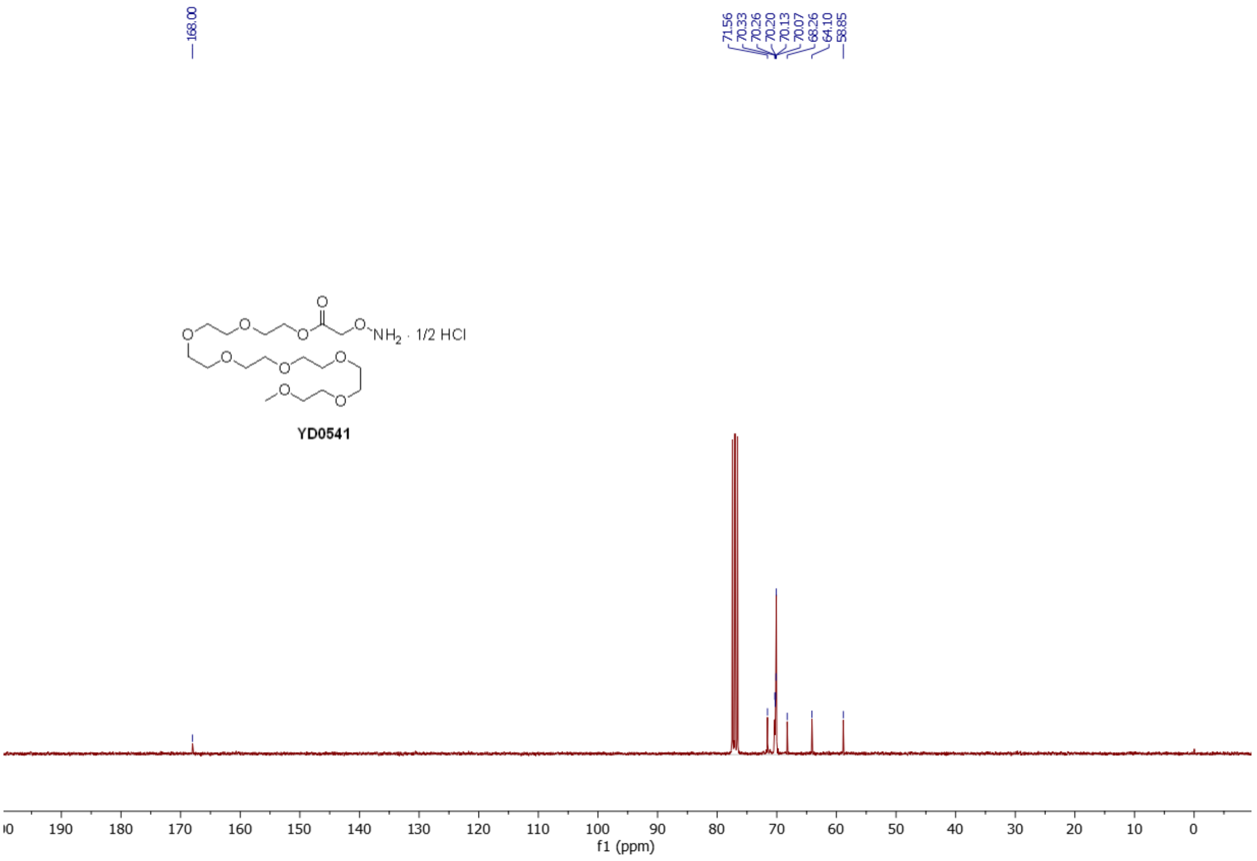

168.00
71.56
70.33
70.26
70.20
70.13
70.07
68.26
64.10
58.85
f1 (ppm)
NH2 · 1/2 HCl
YD0541
